# Time and frequency characteristics of various noninvasive heartbeat sensors

**DOI:** 10.1371/journal.pone.0357922

**Published:** 2026-09-03

**Authors:** Pierre Charlier, Mathieu Jeanne, Maxence Hureau, Julien De Jonckheere

**Affiliations:** 1 CHU Lille, Inserm, CIC 1403 - Centre d’investigation clinique, Lille, France; 2 CHU Lille, Department of Anesthesiology and Critical Care, CHU de Lille, Lille, France; 3 University of Lille, ULR 7365 - GRITA - Groupe de Recherche sur les formes Injectables et les Technologies Associées, University of Lille, Lille, France; 4 University of Lille, ULR 2694 – METRICS, Lille, France; Hubei University, CHINA

## Abstract

Heart rate and cardiac dynamics can be monitored using several physiological signals, each with its own biophysical basis and signal characteristics. However, while individual properties of electrocardiogram (ECG), phonocardiogram (PCG), seismocardiogram (SCG), photoplethysmogram (PPG), and piezoplethysmogram (PiPG) are documented, systematic cross-modal comparisons remain scarce. This study aims to consolidate both the time and frequency domain features of these widely accessible technologies to provide practical guidelines for signal processing and multimodal integration. We simultaneously recorded ECG, PCG, SCG, PPG, and PiPG under standardized resting conditions and aligned all modalities on the ECG R-peak. Results highlight clear consistencies (e.g., reproducible morphology and spectra for ECG, PPG, and PiPG) as well as higher variability in PCG and SCG due to sensor coupling and anatomical factors. Temporal latencies relative to the ECG confirm known physiological conduction delays, while frequency analysis identifies modality-specific bands: ~ 30 Hz for PCG, 11–14 Hz for SCG, and 1–2 Hz for PPG/PiPG. Bandwidth analysis further emphasizes differences in spectral richness across modalities. By presenting these benchmarks in a unified framework, this work addresses the current gap between isolated characterizations and integrated signal knowledge. The outcome is a reference dataset and interpretation map that can guide filtering strategies, feature extraction, and the design of multimodal cardiac monitoring systems.

## Introduction

**Fig 1:**
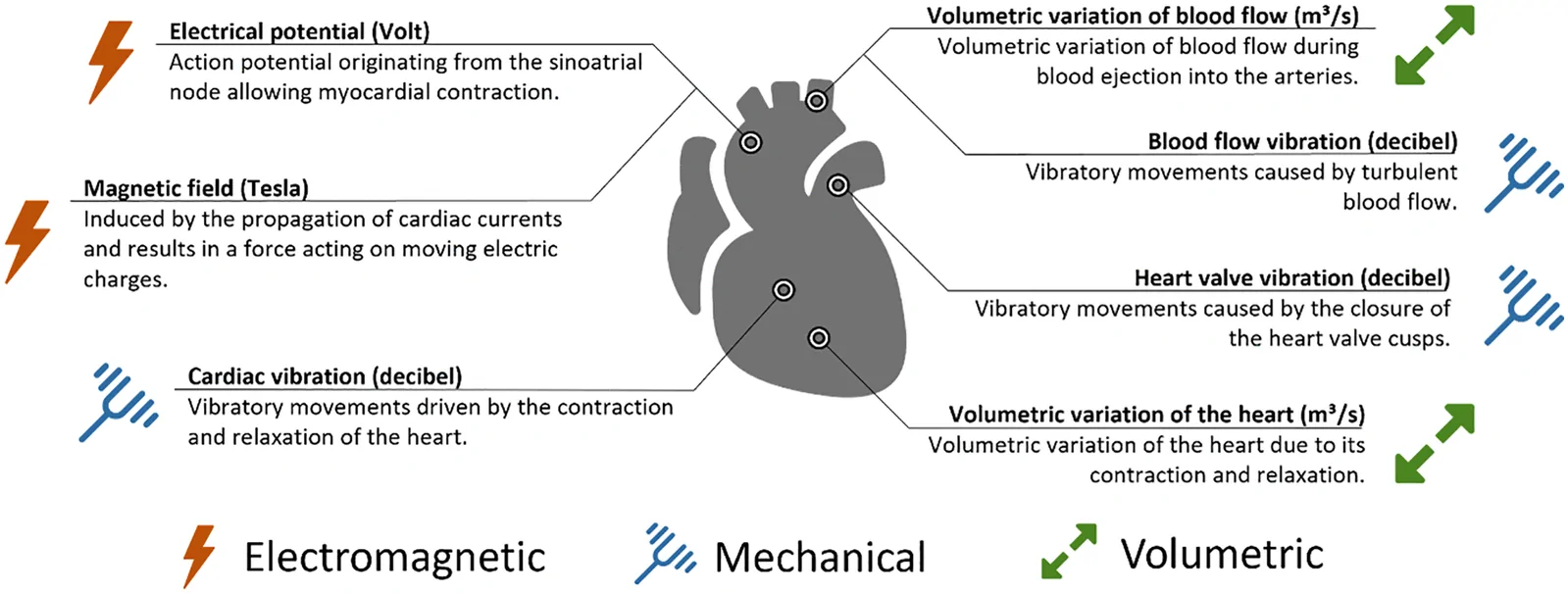
Example of various biophysical principles for heart cycle characterization.

Instantaneous heart rate (HR) in human beings is commonly assessed through physiological signals related to the electrical, magnetic or mechanical characteristics of the heart through its physiological cycle. Several distinct biophysical principles can be implemented for measuring the changes related to the heart cycle: electromagnetic, mechanical and volumetric (Fig 1).

These signal sources can be exploited to detect the cardiac cycle and subsequently measure instantaneous heart rate (HR). For instance, the electromagnetic activity of the heart can be captured through electrocardiography (ECG) or magnetocardiography (MCG); mechanical activity through phonocardiography (PCG), ballistocardiography (BCG), or seismocardiography (SCG); and volumetric changes via Doppler probes (ultrasound or ultra-wideband), photoplethysmography (PPG), impedance cardiography (ICG), or piezoplethysmography (PiPG).

Several cardiac signals are widely accessible, noninvasive and compatible with off-the-shelf sensors, making them well suited for ambulatory and real-world physiological monitoring: ECG, PCG, SCG, PPG, and PiPG, which can be easily integrated into wearable or portable systems due to their low power requirements, compact form factor and simple hardware implementation [1–4].

By contrast, more advanced modalities, such as magnetocardiography, impedance cardiography and Doppler-based techniques offer deeper insights into cardiac cycle dynamics but are generally limited to specialized clinical environments because of their technical complexity, higher cost or lack of miniaturized hardware.

Numerous studies have demonstrated the feasibility of extracting heart rate variability (HRV) from various physiological signals [5,6]. The characterization of their time- and frequency-domain contents has been extensively described for the ECG signal [2,7–12], while description and characteristics of other signals are scarce: PCG [13–15], SCG [16–18], PPG [19,20] and BCG [20–22].

The objective of this study is to present a unified cross-modal characterization of ECG, PCG, SCG, PPG, and PiPG signals acquired simultaneously in human beings under standardized resting conditions and export their temporal and spectral content into a single comparative framework. By exporting their temporal and spectral content into a single comparative framework using identical hardware, this work aims to provide a signal-processing benchmark and physiological comparison that eliminates the inter-system biases often found in separate studies, rather than a hardware-validation study. We aim to provide practical guidelines for filtering, signal analysis and feature extraction in order to contribute to the standardization and optimization of multimodal, noninvasive, wearable cardiac monitoring technologies and facilitate future sensor fusion strategies. Furthermore, while this study focuses on biomedical sensor fusion, these approaches can benefit from and contribute to broader developments in multimodal integrated sensing systems [23–27].

## Methods

### Acquisition system

**Fig 2:**
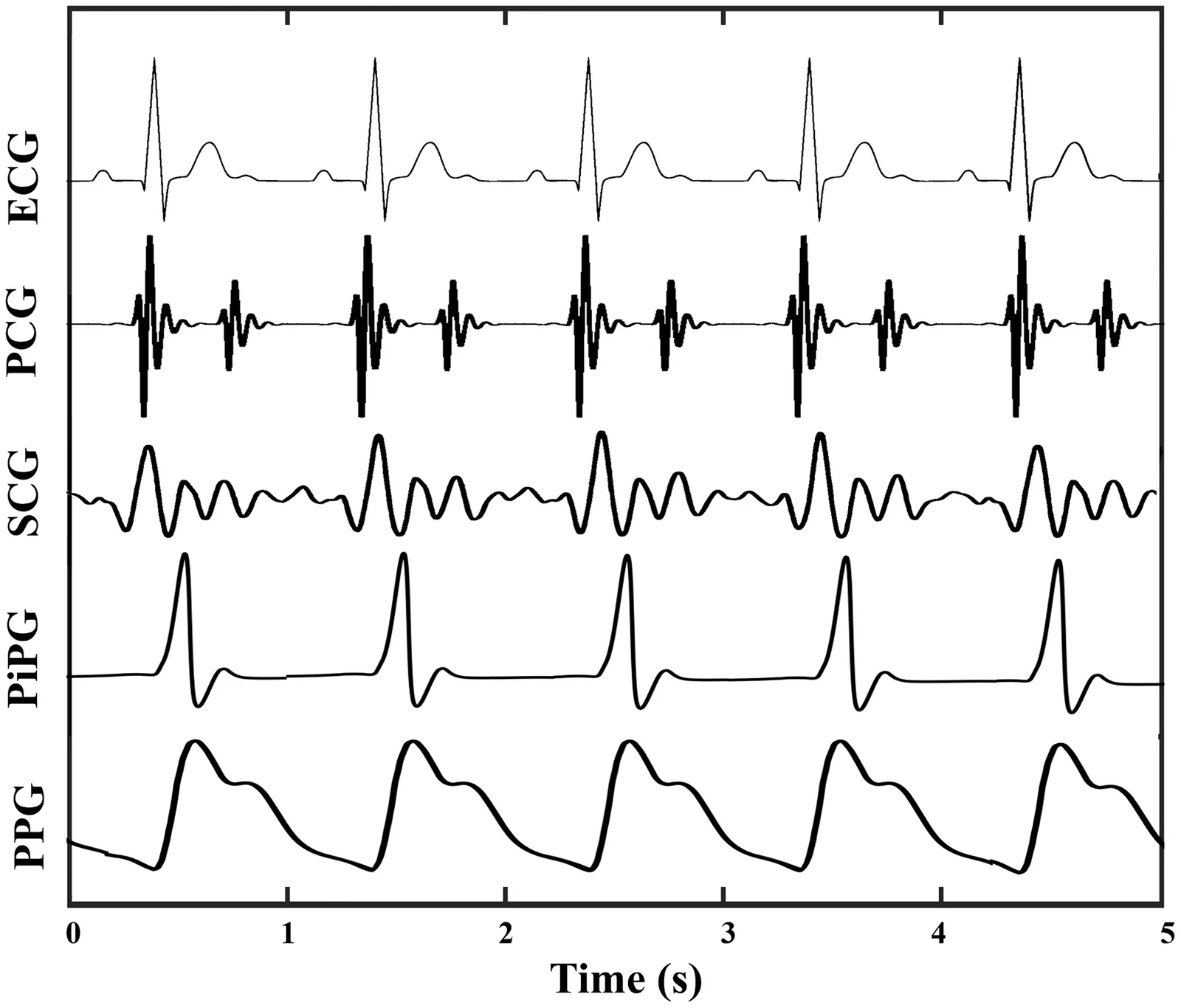
Schematic display of the various waveforms obtained simultaneously: Electrocardiogram (ECG), Phonocardiogram (PCG), Seismocardiogram (SCG), Piezoplethysmogram (PiPG) and Photoplethysmogram (PPG).

Physiological data were acquired using a custom acquisition system developed at the CICIT 1403 (Lille, France) operating at a sampling rate of 1000 Hz and enabling the simultaneous and synchronized recording of five signal modalities: ECG, PCG, SCG, PPG and PiPG (Fig 2).

**ECG –** Electrocardiogram. The cardiac electrical activity was measured using surface Ag/AgCl adhesive electrodes (Red Dot 2670−5, 3M, USA), positioned in a standard Lead I configuration on the thorax. To minimize motion artifacts and enhance signal stability, a driven-right-leg (DRL) circuit was implemented using a bias electrode, effectively reducing common-mode interference, e.g., 50 Hz noise from the power grid. As the system is battery-powered, this further minimizes external power line interference.
**PCG –** Phonocardiogram. Cardiac sounds related to valve closure during the various phases of the cardiac cycle were recorded using an electret microphone (AOM-6738L-R, Projects Unlimited, USA) featuring high sensitivity (−38 dB) and stable frequency response from 50 Hz with an omni-directional polar pattern providing consistent performance in the low-frequency range. The analog audio signal was amplified using a dedicated audio preamplifier (TS472, STMicroelectronics, Switzerland), which also provided the necessary bias voltage for the microphone. This preamplifier provides a high bandwidth of 40 kHz and a very low Equivalent Input Noise (EIN) of 10 nV/√Hz.
**SCG –** Seismocardiogram. Thorax vibrations produced by the beating heart were captured using a high-resolution analog accelerometer (ADXL356, Analog Devices, USA) placed on the lower sternum. This sensor provides a wide frequency response (up to 2400 Hz on the Z-axis) and low noise density (80 μg/√Hz). Although the used accelerometer is three-dimensional, this study focused on the Z-axis in accordance with the orientation of the sensor on the chest.
**PiPG –** Piezoplethysmogram. A piezoelectric sensor (MU0292−1, GETMUSIC, China) was placed on an adjacent finger to the PPG probe. The PiPG sensor captures local mechanical changes caused by arterial pressure waves.
**PPG –** Photoplethysmogram. Blood volume changes at the fingertip were measured using a reflective-mode PPG sensor (MAX30102, Maxim Integrated, USA), integrating red (660 nm) and infrared (880 nm) LEDs along with a photodiode. Only the infrared channel was analyzed for heart rate and heart rate variability analysis, because of its higher performance than red light sensors [1].

**Fig 3:**
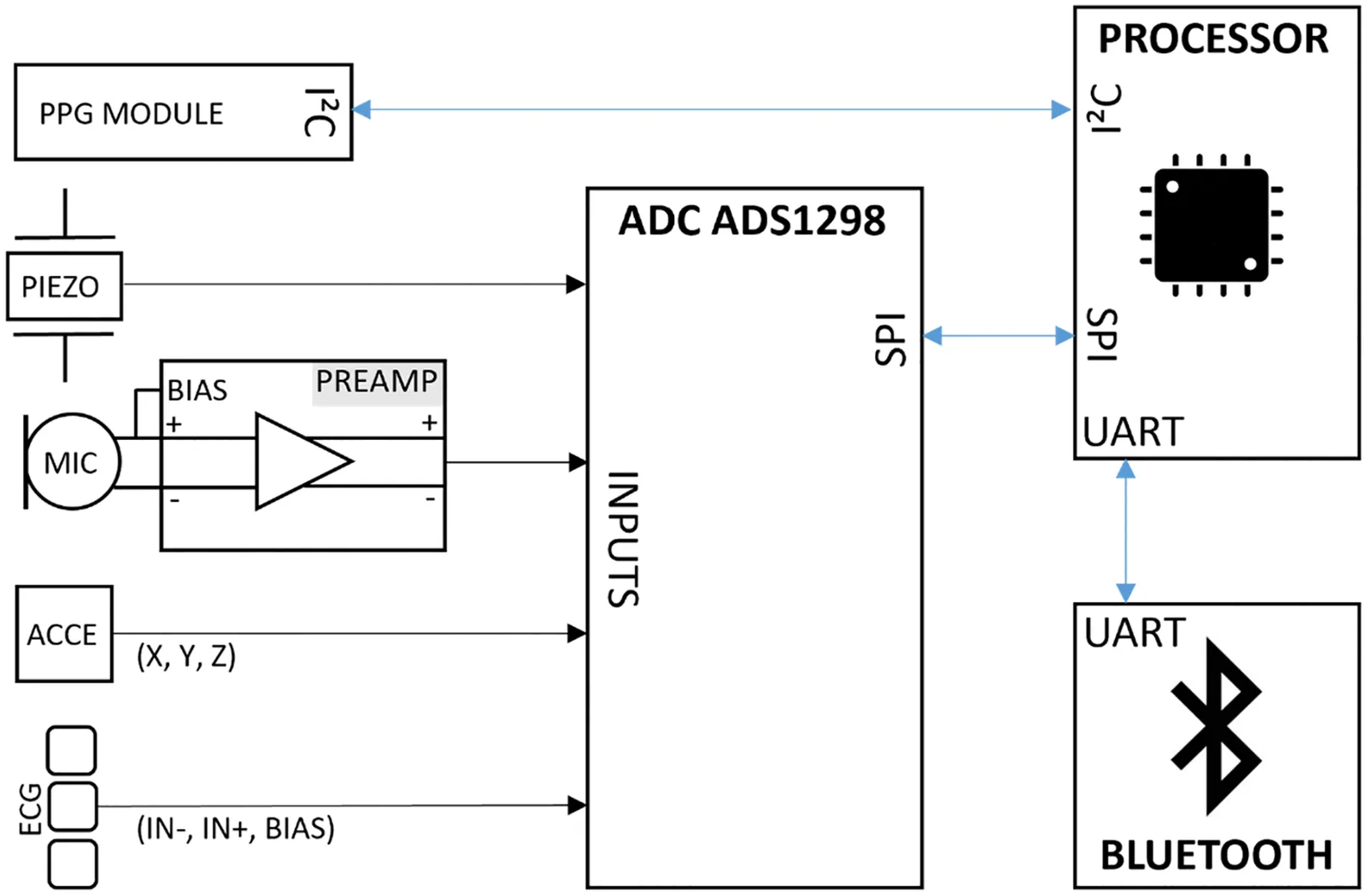
Electronic architecture of the acquisition device: Photoplethysmogram (PPG) module via I2C, Piezoelectric sensor, Microphone (MIC) with preamplifier, Accelerometer (ACCE), and Electrocardiogram (ECG) electrodes connected to an ADS1298 Analog-to-Digital Converter (ADC), interfaced with a Processor and a Bluetooth module.

**Signal digitization and acquisition**. Except for the PPG signal, all analog signals were digitized using a 24-bit digital converter (ADS1298, Texas Instruments, USA) at a 1000 Hz sampling rate. This analog front-end features a highly linear programmable gain amplifier (Integral Nonlinearity of 8 ppm) with available gains of 1, 2, 3, 4, 6, 8, or 12, and an input-referred noise floor of 4 µVPP (at 150 Hz bandwidth, gain = 6). Anti-aliasing is inherently managed by an internal EMI filter (3 MHz bandwidth) and on-chip digital decimation sinc filters. Digitized signals were then transmitted to a SAMD21 microcontroller (Microchip Technology, USA), for signal formatting, which were then transmitted to the Bluetooth communication chip (Fig 3). While a 1000 Hz sampling rate may limit the detailed analysis of high-frequency acoustic harmonics, especially in the PCG signal, it remains more than sufficient for robust heart rate detection and the optimization of noninvasive cardiac monitoring systems, where the primary energy of heartbeat-related signals resides well below the 500 Hz Nyquist frequency.

### Healthy volunteers and data collection

The study was conducted at Lille University Hospital (France). Ethical committee approval was not required as this technical validation involved the retrospective analysis of an anonymized database, with source data originally measured between February 2018 and August 2018. Under French regulations, research not intended to expand medical or biological knowledge is not classified as a clinical study, thereby exempting it from formal ethical oversight. For research purposes, the authors accessed the database on 15 December 2025. The authors had no access to information that could identify individual participants during or after data collection.

Inclusion criteria were: healthy subjects aged from 18 to 60 years old with no history of cardiac or autonomic disease, diabetes or obesity. As the purpose of the study was merely technical, there was no pre-requisite for the healthy volunteers to participate (e.g., medications, fasting, coffee intake, etc.).

**Fig 4:**
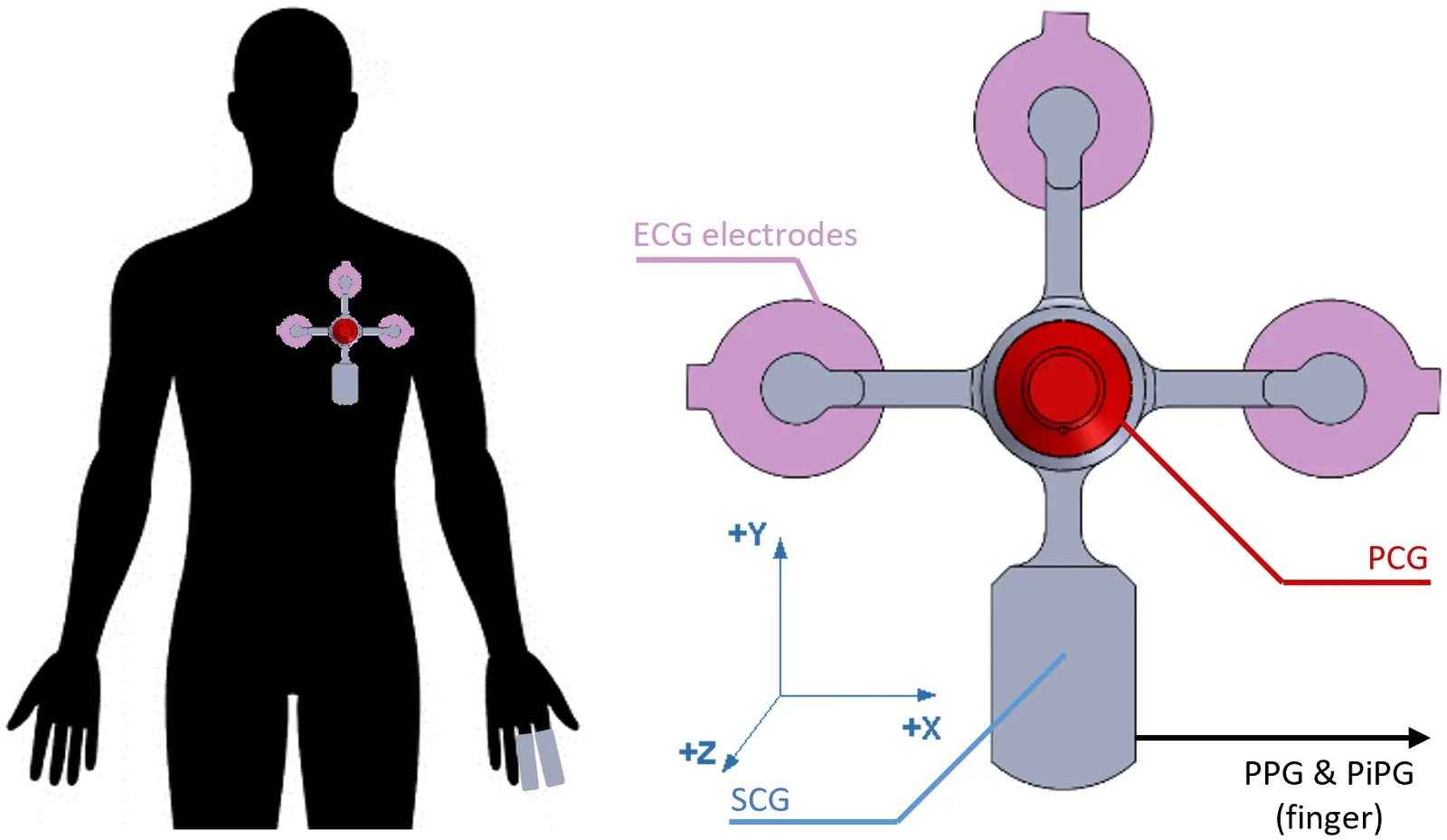
Sensors positioning on the chest wall and two adjacent fingers: Electrocardiogram (ECG) electrodes, Phonocardiogram (PCG) acoustic sensor, and Seismocardiogram (SCG) accelerometer placed on the thorax, with Photoplethysmogram (PPG) and Piezoplethysmogram (PiPG) sensors attached to the fingers.

Upon inclusion, healthy volunteers were asked to choose a comfortable lying position. The total expected duration of the experiment was 10 min, with recording of their cardiac signals of 5 min. The ECG, SCG, and PCG sensors were positioned on the subject’s left chest (Fig 4), with the PCG in contact with the chest skin to record cardiac sounds. A custom-made support was designed to maintain contact between the sensors and the skin (Fig 4) and ensure reproducible inter-individual sensor placement. The PPG and PiPG sensors were placed on two adjacent fingers of the left hand.

### Signal processing

#### Time domain analysis.

Time-domain analysis characterizes the chronology of cardiac events by measuring the latencies between electrical activation (ECG R-peak) and subsequent mechanical or volumetric responses, such as valve closure or pulse waves. It is crucial for assessing waveform morphological consistency and identifying clinical markers like pulse transit time. Peak detection was based on the ECG signal, used as reference because electrical cardiac activity precludes any mechanical changes of the cardiac cycle, such as vibrations, pressure changes or acoustic emissions.

**Fig 5:**
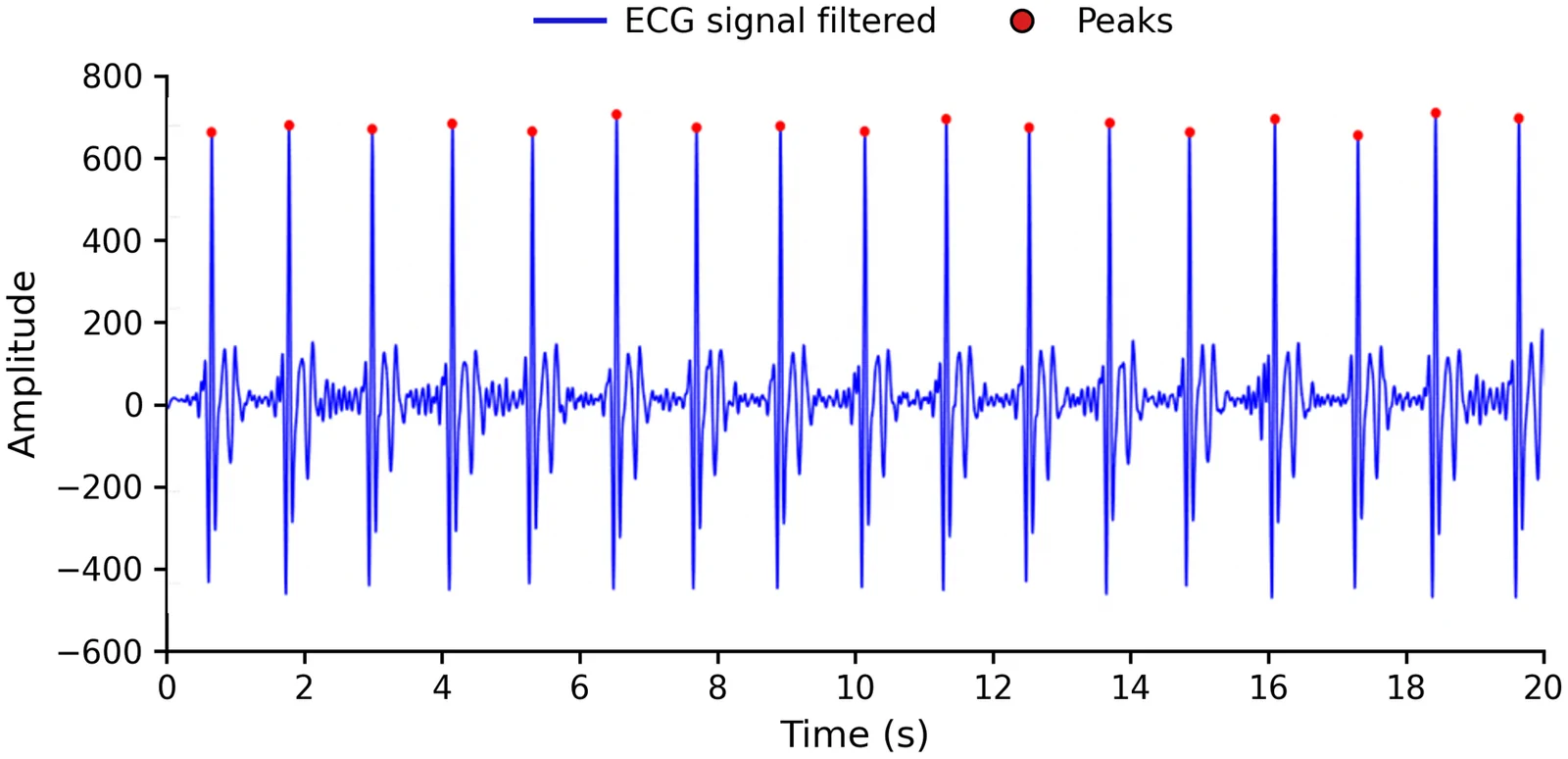
Band-pass filtered ECG (Electrocardiogram) and detected QRS peaks.

The ECG signal was bandpass filtered between 5 and 15 Hz using a 4th-order Butterworth filter [2] (Fig 5); Peak positions were identified using the FindPeaks function in MATLAB (R2018a, The MathWorks, USA), with a refractory period of 600 ms, corresponding to a maximum heart rate of 100 BPM. This relatively short refractory period was chosen as recordings were performed at rest, meaning that subjects were not tachycardic, and because our aim was to favor specificity over sensitivity, i.e., to avoid false detections of QRS complexes and filter out early extrasystoles that might otherwise create inconsistencies across signals. To further ensure the robustness of heart rate estimation, an automated outlier rejection step was implemented, excluding any cardiac cycles with an R-R interval deviating by more than 2.5 standard deviations from the subject’s median.

As the signal was filtered for QRS peak detection, this specific filtering stage was used exclusively for robust R-peak localization. All subsequent time and frequency domain analyses were performed on the raw, unfiltered data to preserve the original waveform morphology and avoid phase-shift induced errors. To ensure temporal accuracy, we refined the detection by searching for a local maximum within a ± 20 ms window around the initial R-peak position.

Based on the detected positions of the ECG R-peak, we extracted the following waves:

The QRS waveform in healthy volunteers is documented as having a duration of less than 120 ms [7], so we extracted the ECG signal over a window of [−60 +60] ms around the R-peak detection.

Phonocardiography (PCG) waves are composed of multiple acoustic waves related to valve changes during the cardiac cycle. We focused on the two primary heart sounds: S1 and S2. S1 is generated by the closure of the mitral and tricuspid valves, marking the start of the systole phase. The S2 sound is produced by the closure of the aortic and pulmonary valves, indicating the beginning of diastole. The S1 peak is expected to occur approximately 10–50 ms after the R-peak of the ECG and to last 100–160 ms, while the S2 peak is generally observed between 280 and 360 ms after the R-peak, and last 80–140 ms [14,15]. We defined PCG extraction windows based on the detected ECG R-peak of [+10 +210] ms for S1 and [+280 +500] ms for S2.

Seismocardiogram (SCG) waves are primarily influenced by myocardial motion and blood flow dynamics rather than valve acoustics [28]. Several characteristic peaks have been identified in SCG signals. In this study, we chose to focus on two SCG components that are temporally aligned with the main PCG heart sounds (S1 and S2). We refer to them as SCG (SCG1) and SCG (SCG2) strictly based on this temporal correspondence, without implying they directly identify specific valve events. Based on these physiological landmarks and to maintain consistency with PCG windowing, we extracted SCG (SCG1) from a window of [+10 +210] ms and SCG (SCG2) [+280 +500] ms after the R-peak.

Piezoplethysmogram (PiPG) waves capture the pulsatile variation in arterial blood volume through the mechanical transduction of pulse at the fingertip, rather than by optical absorption in the case of PPG. PiPG signals exhibit sharper waveforms and higher frequency contents than PPG due to the direct mechanical coupling, making them potentially more sensitive to local vascular dynamics and skin–sensor contact variations. Like PPG, the timing of the PiPG pulse is influenced by the location of the sensor and the subject’s pulse transit time.

Photoplethysmography (PPG) waves do not allow to differentiate between the systolic and diastolic wave. Systolic waves often merge into the diastolic waves, making only a single inflection point (turning point) clearly visible. Furthermore, the timing of PPG waveforms is highly dependent on sensor placement because of the variable pulse transit time (PTT). At the fingertip, the PTT is usually 200–300 ms long, thus occurring after the R-peak [19]. We defined a broad extraction window of [+150 +1350] ms relative to the ECG R-peak, encompassing the whole PPG signal pulse at minimal HR of 50 BPM.

Chosen signal analysis windows for each type of signal, using ECG R-peak as reference, are presented in Table 1.

**Table 1:** Temporal windows for signal peak detection relative to ECG R-peak.

	TSTART (ms)	TSTOP (ms)
**ECG (QRS) – R-peak detection**	−60	+60
**PCG (S1)**	+10	+210
**PCG (S2)**	+280	+500
**SCG (SCG1)**	+10	+210
**SCG (SCG2)**	+280	+500
**PiPG**	+150	+1350
**PPG**	+150	+1350

**Fig 6:**
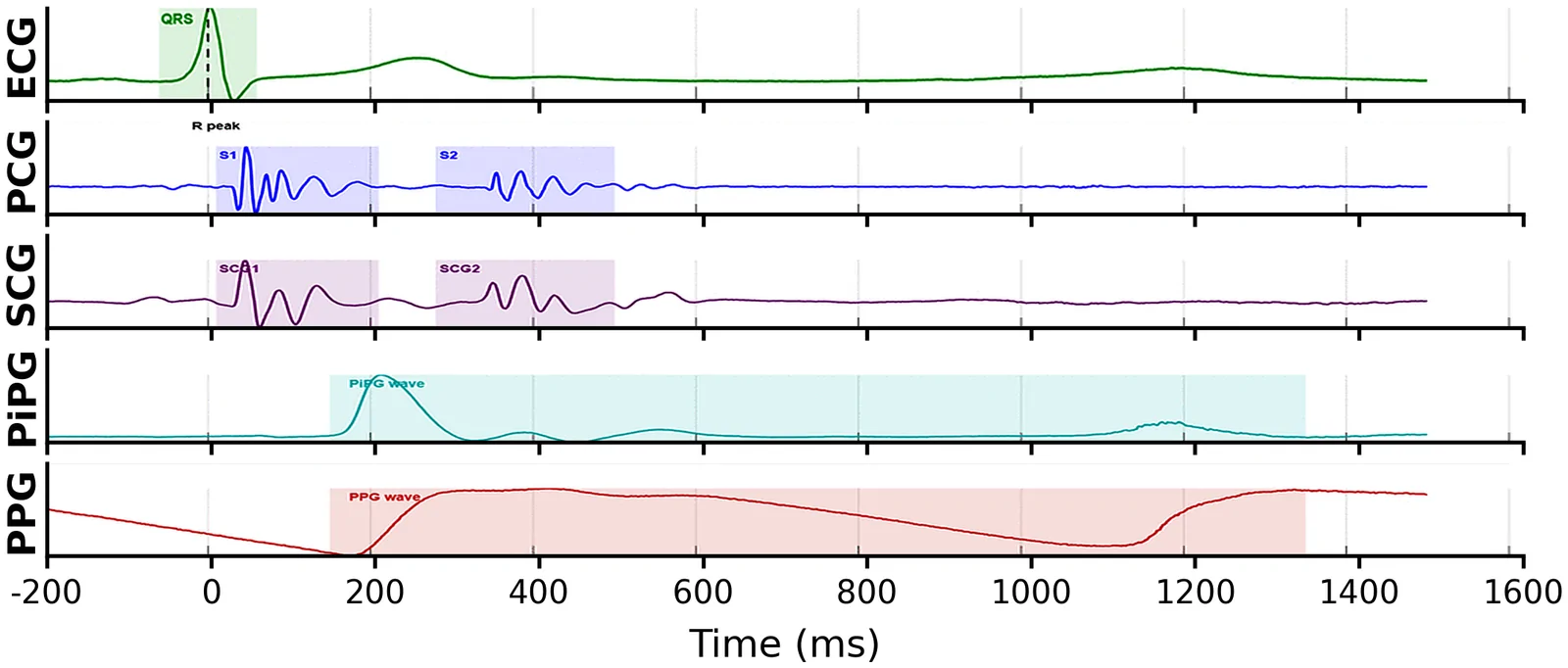
Illustration of peak windows with the different technology: QRS complex and R-peak for Electrocardiogram (ECG), S1 and S2 heart sounds for Phonocardiogram (PCG), SCG (SCG1) and SCG (SCG2) mechanical vibrations for Seismocardiogram (SCG), and the characteristic systolic waves for Piezoplethysmogram (PiPG) and Photoplethysmogram (PPG).

As illustrated in Fig 6, in order to account for inter-individual propagation variability between the electrical activation (QRS complex) and subsequent mechanical or acoustic events, a fixed alignment across subjects would introduce systematic bias. To mitigate this, the wave extraction of each signal was performed around the local maxima within specific time windows for each signal, always by using the ECG R-peak as reference. Then, for each window, a DC subtraction of the wave was applied by subtracting the mean. To control for the potential confounding effect of local peak recentering on waveform alignment, an additional analysis was performed using strict ECG-locked ensemble averaging without local adjustment.

By detecting each pulse wave for each signal over the course of 5 min recordings, a median wave was computed for each signal, and was normalized because signal amplitude is largely determined by sensor-specific gains.

Inter-subject variability was evaluated by comparing each subject’s median waveform to the grand median waveform computed across all volunteers.

For each median waveform modality, the median of all cardiac pulses was computed by synchronizing each individual cardiac cycle on its maximum amplitude within the predefined extraction window. This synchronization facilitates visualization and delay estimation by emphasizing the dominant peak, but may artificially sharpen the resulting waveform. However, this step is necessary to compensate for the intra-individual physiological “jitter” between electrical activation (ECG R-peak) and the subsequent mechanical response (PCG, SCG, or PPG). This variability, influenced by factors such as instantaneous heart rate, autonomic tone, and respiratory phase, would otherwise cause the ensemble averaging process to act as a low-pass filter. Without this local alignment, rapid mechanical transients (such as the S1 sound or SCG waves) would be blurred, misrepresenting their true morphology. This effect is particularly noticeable for signals with high variability in shape and timing.

### Spectral domain analysis

Spectral analysis was performed on each isolated peak window using a Welch’s method–based approach: for each signal pulse wave, the power spectral density (PSD) was estimated with the pwelch function in MATLAB (Hamming window with 50% overlap and a 2048-point FFT). Segments ranged from 200 ms to 400 ms in duration, resulting in an intrinsic frequency resolution of 2.5 to 5 Hz. To remove DC bias, each segment was detrended by subtracting its mean value. The 2048-point FFT was applied to provide a finer spectral interpolation of the frequency domain. Similarly with the time domain analysis, a median power spectral density (PSD) was computed for each signal type using the previously segmented individual cardiac windows. This windowed approach was chosen to isolate the spectral signature of the cardiac waveform morphology from inter-beat noise and baseline fluctuations, ensuring that the extracted features represent the heart pulse itself. Each individual PSD was normalized, and the Pearson correlation coefficient (r) was calculated between each subject’s PSD waves and the group-averaged PSD wave to assess spectral variability.

In addition, to characterize the frequency content of each physiological signal, we extracted two spectral features from the PSD of each subject: the first was the peak frequency, defined as the frequency at which the PSD reaches its maximum power, representing the dominant spectral component of the signal; the second was the –3 dB bandwidth around Peak Frequency, defined as the frequency interval surrounding the peak where the PSD remains above half of its maximum value. The −3 dB bandwidth is an indication of the effective range of usable signal content and is commonly used to guide the design of bandpass filters in signal processing.

### Statistical analysis

For both temporal and spectral domains, intra-individual and inter-individual variability were analyzed using the Pearson correlation coefficient (r) for each signal type. For the inter-individual analysis, the reference waveform was computed using a leave-one-subject-out approach, whereby the participant under evaluation was excluded from the reference average. To assess the significance of the differences between intra- and inter-subject consistency, a Wilcoxon signed-rank test was performed for each modality, with a significance threshold set at p < 0.05. Statistical results are primarily descriptive and are presented as mean ± standard deviation. For reproducibility assessment, 95% confidence intervals (95% CI) were calculated for all Pearson correlation coefficients using the Fisher z-transformation.

No formal multiplicity correction was applied, as this study is primarily descriptive and exploratory, with each modality evaluated independently against its reference to establish baseline characteristics.

## Results

**Fig 7:**
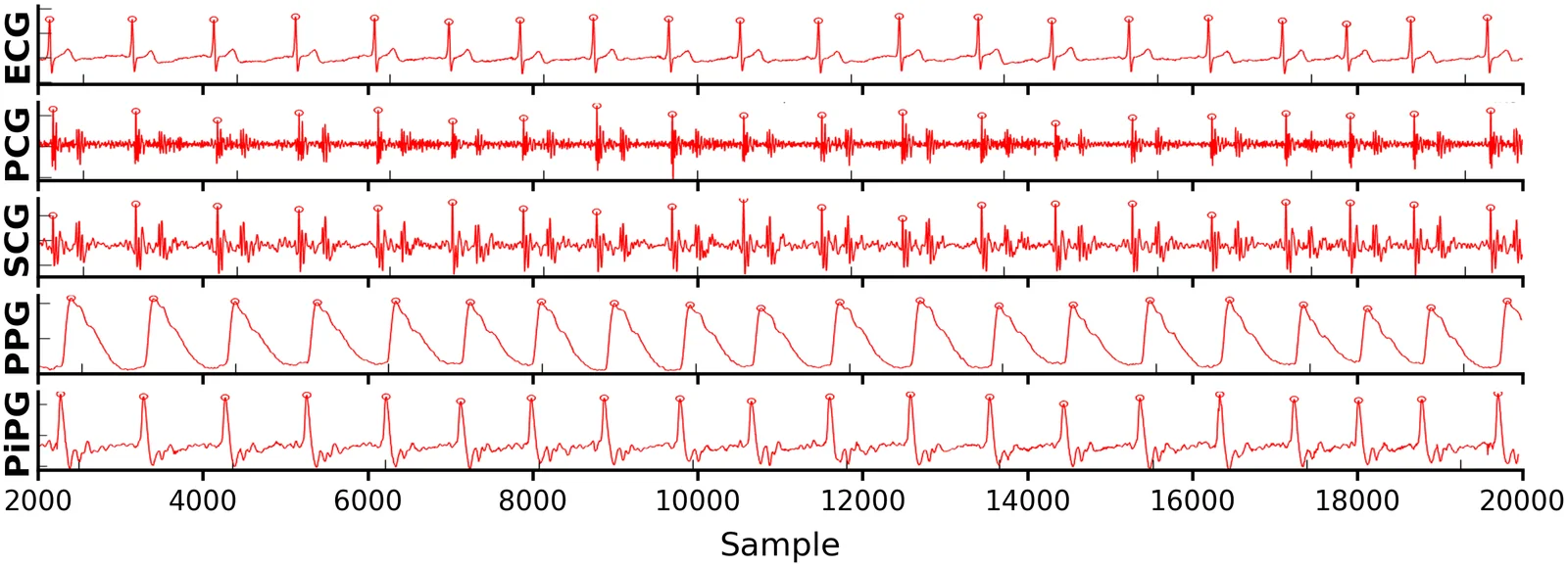
Example of recorded signals showing peak detection for Electrocardiogram (ECG), Phonocardiogram (PCG), Seismocardiogram (SCG), Piezoplethysmogram (PiPG) and Photoplethysmogram (PPG).

A total of 20 healthy adult volunteers (10 males and 10 females), aged 24–52 years with a median age of 29, were included in the study. The subjects’ heart rates remained stable throughout the recordings, with a mean of 62.3 BPM (range: 57.1–71.4 BPM). Across the 20 recordings, 6360 cardiac cycles were detected. The automated RR outlier rejection procedure excluded 171 cycles (2.7% overall), with a maximum rejection rate of 12.5% in a single recording. Tolerance of the recording period was good, with no side effect recorded. All subjects rested during the recording period, which lasted 5 min in all cases. All recorded signals were of good quality, and were all processed as described in the Method section. An example of a recording can be found in Fig 7.

### Time domain analysis

**Fig 8:**
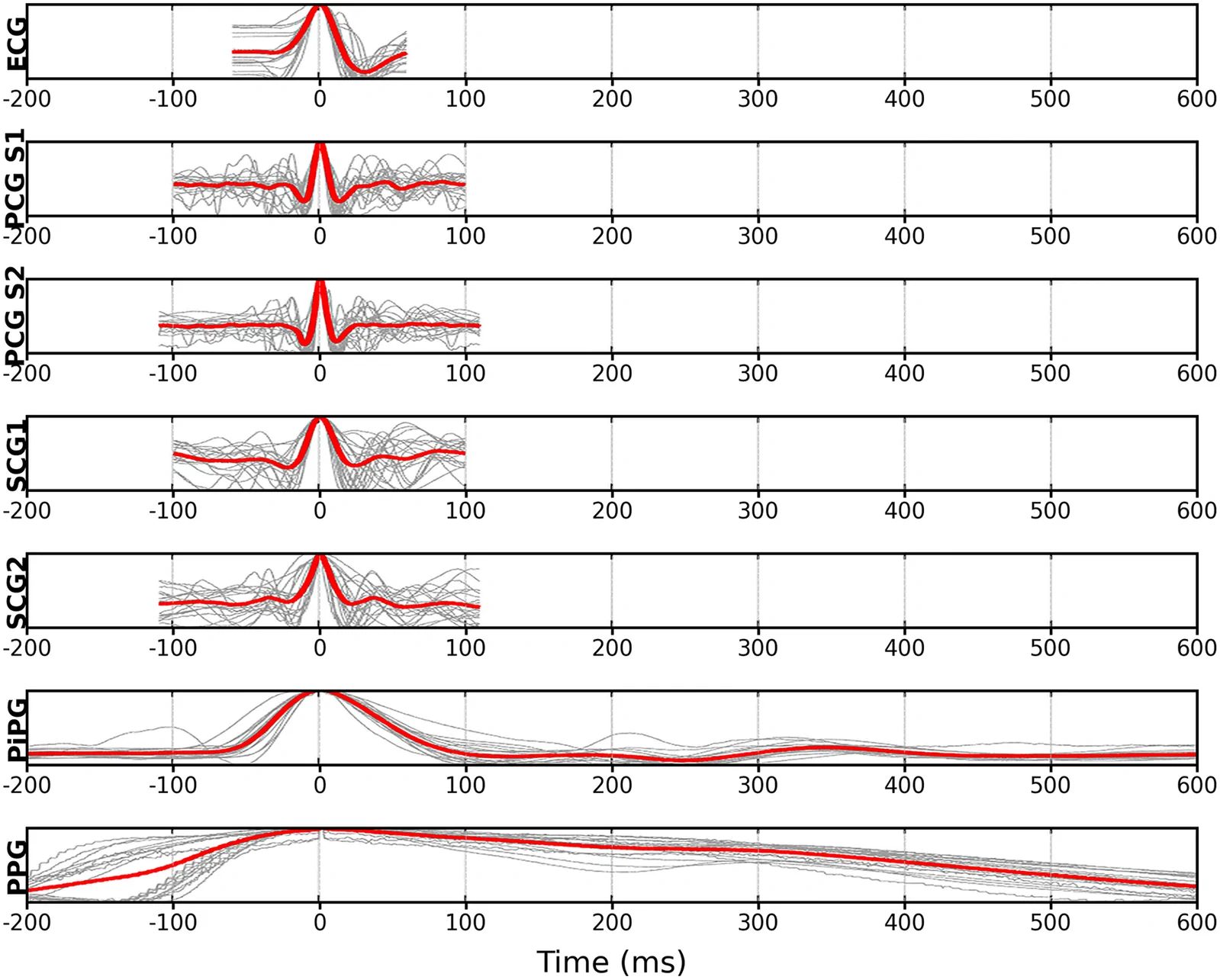
Individual subject waveforms (grey) and group median waveforms (red) for Electrocardiogram (ECG), Phonocardiogram (PCG), Seismocardiogram (SCG), Piezoplethysmogram (PiPG) and Photoplethysmogram (PPG). Time 0 corresponds to the ECG R-peak.

Fig 8 shows the individual extracted waveforms (grey lines) and the corresponding median waveform (red line) for ECG, PCG (S1), PCG (S2), SCG (SCG1), SCG (SCG2), PiPG and PPG. All waveforms are centered on 0, which is defined as the R-wave of the ECG. The time delays specific to each signal (as discussed in the Method section) are artificially nullified, so as to show the impact of each heart beat on each signal independently of its physical nature. All signals are presented in a [−200 +600] ms window.

The quantitative results of morphological similarity are summarized Table 2. High consistency was observed for ECG (r = 0.90 ± 0.08), PPG (r = 0.90 ± 0.05), and PiPG (r = 0.96 ± 0.05), indicating low inter-individual variation. PCG signals exhibited moderate variability, with S1 and S2 showing r = 0.75 ± 0.10 and r = 0.79 ± 0.10, respectively. SCG waveforms were more variable (r < 0.7). Intra-subject correlations were higher than inter-subject correlations for ECG and SCG signals, highlighting their consistency within individuals. In contrast, PPG and PCG signals showed comparable or even higher inter-subject correlations.

**Table 2:** Wave similarity quantified using Pearson correlation.

wave type	Pearson r		p
	Intra individual ^a^	Inter individual ^a^	
**ECG (QRS)**	0.97 ± 0.04 [95% CI: 0.95–0.99]	0.90 ± 0.08 [95% CI: 0.86–0.94]	<0.001
**PCG (S1)**	0.70 ± 0.13 [95% CI: 0.64–0.76]	0.74 ± 0.11 [95% CI: 0.69–0.79]	<0.001
**PCG (S2)**	0.67 ± 0.16 [95% CI: 0.60–0.74]	0.77 ± 0.11 [95% CI: 0.72–0.82]	<0.001
**SCG (SCG1)**	0.83 ± 0.13 [95% CI: 0.77–0.89]	0.66 ± 0.10 [95% CI: 0.61–0.71]	<0.001
**SCG (SCG2)**	0.77 ± 0.14 [95% CI: 0.70–0.84]	0.69 ± 0.12 [95% CI: 0.63–0.75]	<0.001
**PiPG**	0.96 ± 0.05 [95% CI: 0.94–0.98]	0.96 ± 0.05 [95% CI: 0.94–0.98]	<0.001
**PPG**	0.86 ± 0.13 [95% CI: 0.80–0.92]	0.90 ± 0.05 [95% CI: 0.88–0.92]	<0.001

^a^Results presented as mean ± standard deviation [95% CI].

Temporal delays between ECG R-peaks and the following peaks of each signal modality are presented in Table 3. As expected, S1 occurred around 77 ms post-R-peak and S2 near 400 ms, consistent with the timing of valve closures. SCG1 and SCG2 showed similar delays (around 80 ms and 400 ms, respectively), confirming their alignment with PCG events. The PiPG signal peaked around 250 ms post-R, while the PPG peaked around 380 ms.

**Table 3:** Temporal delay between the ECG R-wave and Peak Detection.

Wave type	Mean delay Inter individual (milliseconds) ^a^	Mean of STD delay Intra individual (milliseconds) ^b^
**PCG (S1)**	76.81 ± 17.76	±27.65
**PCG (S2)**	399.87 ± 25.47	±25.29
**SCG (SCG1)**	80.15 ± 27.70	±19.01
**SCG (SCG2)**	393.87 ± 28.94	±36.03
**PiPG**	251.56 ± 21.58	±17.53
**PPG**	378.09 ± 47.43	±48.24

^a^Results presented as mean ± standard deviation.

^b^Results presented as ± standard deviation.

While local peak recentering significantly improved the temporal alignment and morphological consistency across subjects, strict ECG-locked alignment without recentering resulted in a substantial decrease in correlation coefficients (detailed in Supporting Information), confirming the presence of physiological timing jitter.

### Spectral domain analysis

**Fig 9:**
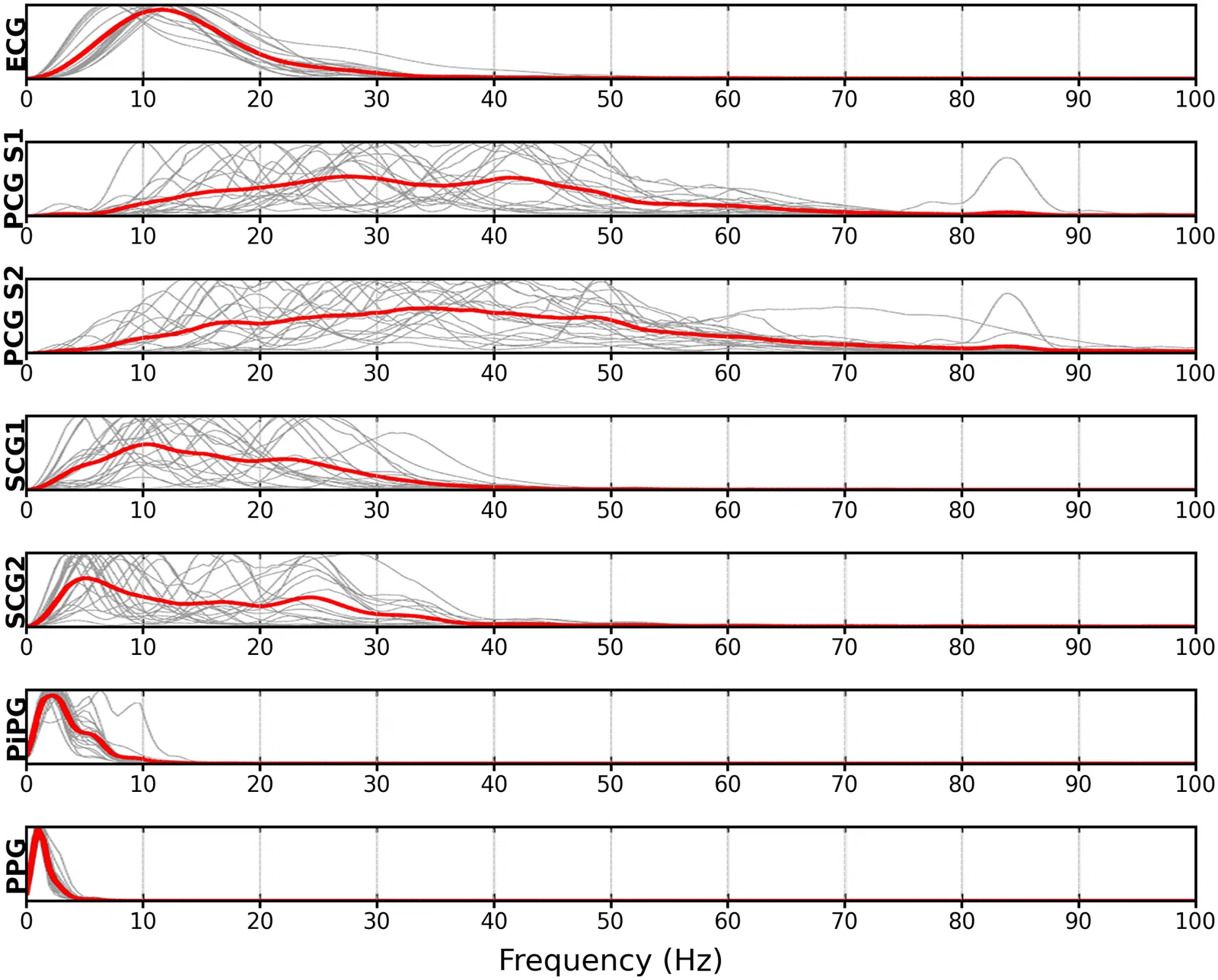
Power Spectral Density (PSD) analysis of each signal modality. Individual subject PSD curves are shown in grey, while the solid red line represents the group median PSD waveform for Electrocardiogram (ECG), Phonocardiogram (PCG), Seismocardiogram (SCG), Piezoplethysmogram (PiPG), and Photoplethysmogram (PPG). The x-axis represents the frequency in Hertz (Hz).

Fig 9 shows the individual extracted PSD (grey lines) and the corresponding median PSD (red line) for ECG, PCG (S1), PCG (S2), SCG (SCG1), SCG (SCG2), PiPG and PPG in spectrum windows of [0 + 100] Hz.

The inter-subject similarity of the spectral signatures is presented in Table 4. High consistency was observed for ECG (r = 0.96 ± 0.04), PiPG (r = 0.97 ± 0.06), and PPG (r = 0.98 ± 0.02) PSDs, indicating strong reproducibility of dominant frequency content across individuals. PCG and SCG signals showed slightly lower inter-subject correlations, ranging from r = 0.78 ± 0.11 (PCG (S1)) to r = 0.83 ± 0.12 (PCG (S2)), suggesting greater variability of their spectral content. Intra-subject correlations were consistently higher in all signal modalities than inter-subject correlations, with ECG, PiPG, and PPG again showing the highest intra-individual reproducibility (r ≥ 0.97).

**Table 4:** PSD peaks similarity quantified using Pearson correlation.

PSD Peak type	Pearson r		p
	Intra individual ^a^	Inter individual ^a^	
**ECG QRS PSD**	0.99 ± 0.02 [95% CI: 0.96–1.00]	0.96 ± 0.04 [95% CI: 0.94–0.98]	<0.001
**PCG (S1) PSD**	0.84 ± 0.10 [95% CI: 0.63–0.94]	0.78 ± 0.11 [95% CI: 0.73–0.83]	<0.001
**PCG (S2) PSD**	0.81 ± 0.14 [95% CI: 0.57–0.92]	0.83 ± 0.12 [95% CI: 0.77–0.89]	<0.001
**SCG (SCG1) PSD**	0.89 ± 0.14 [95% CI: 0.74–0.96]	0.84 ± 0.13 [95% CI: 0.78–0.90]	<0.001
**SCG (SCG2) PSD**	0.88 ± 0.08 [95% CI: 0.71–0.95]	0.82 ± 0.12 [95% CI: 0.76–0.88]	<0.001
**PiPG PSD**	0.98 ± 0.04 [95% CI: 0.94–0.99]	0.96 ± 0.06 [95% CI: 0.93–0.99]	<0.001
**PPG PSD**	0.97 ± 0.03 [95% CI: 0.91–0.99]	0.97 ± 0.03 [95% CI: 0.96–0.98]	<0.001

^a^Results presented as mean ± standard deviation [95% CI].

Spectral features are presented in Table 5. The ECG QRS exhibited a peak frequency around 11 Hz, with a relatively narrow bandwidth (6–18 Hz), consistent with the known sharpness of the QRS complex. PCG signals (S1 and S2) displayed a broader and higher-frequency content, peaking around 30–31 Hz, which is in line with their acoustic nature. SCG components showed intermediate frequencies, between 11 and 14 Hz, but with a wider variability, which can be probably explained by sensor positioning, partial signal loss and tissue coupling.

**Table 5:** PSD peaks frequency and −3 dB bandwidth.

PSD Peak type	Peak frequency (Hz) ^a^	−3 dB bandwidth ^b^
**ECG QRS**	10.99 ± 2.25	[5.98 ± 1.75; 17.87 ± 2.20]
**PCG (S1)**	31.49 ± 11.03	[26.22 ± 10.60; 37.23 ± 12.46]
**PCG (S2)**	30.49 ± 10.02	[24.76 ± 9.28; 40.04 ± 12.07]
**SCG (SCG1)**	14.50 ± 6.75	[9.72 ± 6.28; 19.21 ± 7.40]
**SCG (SCG2)**	11.16 ± 7.46	[7.71 ± 6.46; 15.23 ± 8.64]
**PiPG**	2.39 ± 0.98	[0.88 ± 0.20; 5.03 ± 1.70]
**PPG**	1.29 ± 0.29	[0.24 ± 0.25; 2.51 ± 0.36]

^a^Results presented as mean ± standard deviation.

^b^Results presented as [Min ± standard deviation; Max± standard deviation].

In contrast, PiPG and PPG signals exhibited a lower spectral content with peak frequencies of 2.4 Hz and 1.3 Hz respectively, which reflects their fluid origin, with arterial blood content considered as a homogeneous fluid from the heart to the capillaries where PiPG and PPG signals are acquired. The relatively slow nature of pulse waveforms is hence characterized by lower peak frequencies than other signal modalities. PiPG consistently showed a slightly broader spectrum than PPG, likely because of its mechanical nature and high-frequency sensitivity.

## Discussion

In the temporal domain, a significant waveform similarity was observed between ECG and PiPG signals (Pearson’s r > 0.9), supporting their robustness and morphological consistency across individual beats and between subjects. The PPG signal also exhibited high inter-individual similarity (r > 0.9), but slightly lower intra-individual similarity (r = 0.86 ± 0.13). This discrepancy is unlikely to be explained by sensor positioning alone, as PiPG and PPG share the same finger placement. Rather, we believe it may be attributed to the low-frequency content and higher sensitivity of the PPG signal to slow baseline fluctuations and DC drift. These three signals demonstrate however a high morphological agreement, particularly between subjects.

In contrast, PCG and SCG signals showed lower intra- and inter-subject correlations (r ≈ 0.67–0.83), suggesting a greater variability in waveform morphology, both within and between individuals. These differences may reflect the influence of acquisition conditions and sensitivity to anatomical variability. This is consistent with the findings of Silva et al. [19] who leveraged simultaneous recordings of ECG and pulsatile signals to improve heartbeat detection. Their work emphasized the importance of waveform alignment and signal fusion across modalities, highlighting the shared temporal dynamics inherent to these physiological signals. The greater variability observed in PCG signal underscores its sensitivity to individual anatomical differences and sensor coupling conditions, a phenomenon previously described by Shino et al. [14] who demonstrated that sound pressure levels and signal characteristics in phonocardiograms can vary significantly from one subject to another, posing challenges for establishing diagnostic thresholds. Similar findings have been reported for SCG signals; Centracchio et al. [17] and Sørensen et al. [18] emphasized the significant inter-subject variability in morphology and fiducial point identification.

In our study, the peaks were searched within ranges reported in the literature and measured delays from the ECG R-peak were found within expected latencies of mechanical cardiac events and peripheral circulation. PCG components S1 and S2 were respectively located around 70 ms and 400 ms after the R-peak, as reported by Ahlström et al. [15]. However, a marked inter-subject variability was observed in PCG morphology and amplitude. This phenomenon has been previously highlighted by Shino et al. [14]. SCG (SCG1) and SCG (SCG2) peaks in SCG signals typically occur approximately 75 ms and 390 ms after the ECG R-peak, respectively, as consistently reported by Sørensen et al. [18] and Centracchio et al. [17]. While PPG and PiPG delays correspond to peripheral pulse propagation, dominant systolic components occur after the R-peak at approximately 250 ms for PiPG and 390 ms for PPG, as reported by Silva et al. [19].

The high variance observed in individual mean delays for PCG and SCG signals can be explained by occasional peak misdetections caused by motion artifacts or low signal-to-noise ratios in some subjects.

In the spectral domain, high PSD similarity was observed among ECG, PiPG, and PPG signals (Pearson’s r > 0.9), indicating stable and reproducible spectral profiles both within and across subjects. The PPG PSD analysis appeared more stable in the intra-individual frequency domain than the temporal domain analysis. This may be explained by the fact that PPG spectra are dominated by the fundamental heart rate frequency and its intrinsically low-frequency content. Similarly to the temporal results, PCG and SCG signals exhibited lower spectral similarity across subjects, with inter-individual correlation coefficients ranging from r = 0.79 to 0.84.

The frequency analysis shows clear differences between signal modalities.

- ECG signals present a dominant spectral content in the [6–18] Hz windows, with a peak at 11 Hz, corroborating the findings of several authors for optimal QRS detection performance (Moraes [9] [9–30] Hz; Benitez [10] [8–20] Hz; Elgendi [12] [8–20] Hz; Sahambi [8] [3–40] Hz; Cuiwei [11] [8–58.5] Hz; Pan & Tompkins [2] [5–15] Hz; Tereshchenko [29] [8–50] Hz).
- PCG signals present broader spectral content, extended in the [10–40] Hz window, with peaks typically near 30 Hz. While Yoganathan et al. [13] and Ahlström et al. [15] reported spectral windows of [10–140] Hz, we did not identify a significant frequency peak difference between S1 and S2. This similarity may be linked to the resting state of the participants, where the primary vibrations of both sounds often overlap. Furthermore, the recording site and the specific acoustic coupling might naturally emphasize the lower-frequency components of the cardiac cycle. While the signal may contain higher-frequency energy, the dominant power in our measurements remains centered around 30 Hz, likely reflecting the fundamental mechanical resonance of the chest and heart system.
- SCG signals present a limited frequency range of [7–15] Hz in our study, narrower than the one reported by (Sørensen [18] [0.05–90] Hz; Centracchio [17] [0.5–40] Hz; Pustozerov [16] [0.5–25] Hz). No significant spectral differences were observed between SCG (SCG1) and SCG (SCG2) indicating that the mechanical vibrations of both systolic and diastolic cardiac activities reside within the same resonance band in this study.
- PiPG and PPG signals present spectral distribution in the [1–2] Hz window, consistent with their hemodynamic origin and beat-to-beat periodicity. Other authors found similar results: Elgendi et al. [30] [0.5–4] Hz, Shao et al. [20] [<10] Hz, Silva et al. [19] [<8] Hz. The frequency profile of PiPG is moderately wider than the one of PPG, which is probably due to mechanical transduction and an enhanced sensitivity to higher frequency perturbation components like movement.

Several methodological limitations must be considered. First, the frequency content of the recorded signals is inherently influenced by the characteristics of the acquisition hardware, including sensor frequency response and analog front-end characteristics. For PCG acquisition, the manufacturer specifies the operating frequency range of the electret microphone from 50 Hz to 16 kHz, without providing a calibrated response below 50 Hz. The microphone is conditioned using a TS472 low-noise audio preamplifier. Internal characterization of the complete PCG acquisition chain (microphone, preamplifier, and ADC) demonstrated that the system remains sensitive down to approximately 15 Hz, although the response is not fully linear in this range. Consequently, because no formal calibration protocol or transfer-function curve was established below 50 Hz for this specific setup, the reported 30 Hz peak for the PCG signal must be considered explicitly device-specific and qualitative. For SCG acquisition, the ADXL356 accelerometer provides a bandwidth of up to 2400 Hz on the Z-axis.

In our study, this was mitigated by selecting components with well-documented responses and by applying minimal preprocessing. Second, sensor placement (particularly for ECG electrodes) can influence waveform projection and spectral distribution; electrodes were consistently positioned along the Lead I axis to ensure standardization. Third, the chosen sampling rate of 1000 Hz represents a balance between spectral fidelity and embedded system compatibility. While this rate fulfills the Nyquist criterion for the primary energy of heartbeat signals, it may underrepresent high-frequency harmonics, especially in PCG and SCG.

However, this sampling frequency remains sufficient for the study’s primary objective: optimizing heartbeat detection and characterizing the dominant spectral components of multimodal monitoring systems. Additionally, data were collected under lying (resting) conditions in order to reduce motion artifacts; while this improves signal stability, it limits the extrapolation of noise characteristics to clinical, ambulatory or dynamic settings. Motion artifacts differentially affect these modalities: PPG is primarily sensitive to low-frequency baseline drifts from optical coupling shifts, whereas PiPG is susceptible to a broader frequency range of mechanical noise and friction due to the piezoelectric sensor’s high sensitivity. Finally, waveform morphology (especially for PPG and PiPG) is influenced by arterial properties and measurement location. Fingertip signals tend to exhibit smooth and delayed profiles due to peripheral vascular compliance and pulse transit time variability, which may blur fine features and contribute to inter-subject variability. The study population was limited to 20 healthy adults (24–52 years). While this modest sample size restricts clinical generalizability, it is statistically sufficient for a descriptive technical analysis, as the study’s robustness relies on the high volume of analyzed cardiac cycles rather than the number of subjects. In older populations or those with cardiovascular pathologies, factors like increased arterial stiffness or valvular diseases would significantly alter pulse transit times and the spectral signatures of mechanical signals (SCG/PCG). Additionally, individual factors such as gender and Body Mass Index (BMI) were not accounted for; differences in chest wall anatomy and higher BMI may increase the attenuation of mechanical vibrations through adipose tissue, potentially affecting the morphological clarity of SCG and PCG recordings. Consequently, the reported delays should be considered as representative ranges for a healthy adult population rather than absolute physiological constants.

Furthermore, it is important to acknowledge that the high correlation coefficients (Pearson’s r) reported for waveform similarity are partly influenced by our preprocessing pipeline.

By normalizing amplitudes and performing local temporal alignment to the peak, we intentionally prioritized the assessment of morphological consistency for detector design. However, this approach mechanically inflates the correlation by removing the variance associated with absolute signal magnitude and physiological timing jitter. Without these normalization and alignment steps, the resulting r values would likely be lower, reflecting the inherent impact of gain fluctuations and intra-individual timing variability on raw signal reproducibility. A fixed 600 ms refractory period was used for R-peak detection, which was suitable for our resting subjects. However, for ambulatory or exercise settings where HR exceeds 100 BPM, this period should be dynamically adapted—for instance, by scaling it to 50–60% of the preceding average R-R intervals. This would prevent QRS omissions during tachycardia while maintaining noise rejection during bradycardia.

These extracted spectral features offer practical guidance for signal processing. While the frequency windows identified above define the core signal energy, they also dictate specific filter designs: broader bandpass filters are required for PCG to preserve S1 and S2 morphology, whereas narrower filters centered around 10–15 Hz are more suitable for SCG.

Furthermore, the very low-frequency profiles of PiPG and PPG make them well-suited for beat detection and HR estimation, though they may theoretically limit sensitivity to high-frequency HRV components, such as respiratory modulation. It should be noted that the dominant 1–2 Hz spectral content observed in these modalities reflects the participant’s fundamental heart rate rather than an intrinsic modality-specific bandwidth; however, because these signals represent hemodynamic pulse waves, this range inherently captures the primary physiological information required for pulse-based monitoring. However, these constraints were addressed in our previous research [5], which demonstrated that despite these spectral characteristics, both PPG and PiPG provide reliable HRV indices when compared on a single dataset. Anatomical dimensions such as subjects’ height or arm length were not recorded or used to normalize the temporal delays of peripheral signals. Since Pulse Transit Time (PTT) is intrinsically dependent on the distance between the heart and the measurement site (the fingertip), these dimensions contribute to the inter-subject variability observed in PPG and PiPG peak latencies. Consequently, the reported delays should be considered as representative ranges for an adult population rather than absolute physiological constants.

## Conclusion

Overall, our study provides a comprehensive time- and frequency-domain characterization of multiple physiological signals synchronized on the ECG R-peak, including ECG, PCG, SCG, PiPG, and PPG, within a small cohort of healthy adults at rest. Consequently, our claims regarding general modality characteristics and standardization remain restricted to this specific population. Our results confirm that electrically and hemodynamically driven signals (ECG, PiPG, PPG) exhibit strong inter-individual consistency, whereas mechanically and acoustically driven signals (PCG, SCG) show greater sensitivity to anatomical variability and sensor placement.

From an application perspective, ECG remains the reference modality for HRV analysis, due to its high temporal precision and broad spectral content. PiPG may serve as a suitable mechanical alternative in scenarios where ECG acquisition is impractical or undesirable. For straightforward heart rate (HR) estimation, PPG remains a practical and robust solution, offering high inter-subject reproducibility and easy implementation, despite slightly lower intra-subject morphological consistency.

These findings support the implementation of signal-specific filtering strategies and guide signal selection based on application needs. They also reinforce the value of peripheral hemodynamic signals in HRV analysis under appropriate conditions, as long as their spectral limitations are accounted for.

## Supporting information

S1 File Minimal data set. This file contains multiple sheets detailing the underlying data for intra-individual wave delays, power spectral density (PSD), wave similarity, and detection counts.
(XLSX)
