# Supplementary material for: Time and frequency characteristics of various noninvasive heartbeat sensors
Source: PLoS One. 2026 Sep 3;21(9):e0357922. doi: 10.1371/journal.pone.0357922 (PMC13541146; doi:10.1371/journal.pone.0357922)
Supplement: Supplementary file 1 [file pone.0357922.s003.docx]

| Pierre Charlier  CHU Lille, CIC 1403  Lille, France  [pierre1.charlier@chu-lille.fr](mailto:pierre1.charlier@chu-lille.fr) | April 27, 2026 |
| --- | --- |

Subject: Submission PONE-D-26-10913

Dear Dr. Ming Zhang and Reviewers,

We would like to thank the editorial board and the reviewers for their constructive comments and suggestions regarding our manuscript. We have carefully addressed each point raised and have revised the manuscript accordingly.

Below is a point-by-point response to the reviewers' comments.

Please note that all line numbers mentioned in our responses refer to the "Revised Manuscript with Track Changes" version of the document

**Response to Journal Requirements**

1) Please ensure that your manuscript meets PLOS ONE's style requirements, including those for file naming.

We have updated the manuscript to strictly adhere to PLOS ONE’s formatting guidelines, including section headings, font requirements, and file naming conventions. All figures have been tested with the NAAS tool.

2) You indicated that ethical approval was not necessary for your study. We understand that the framework for ethical oversight requirements for studies of this type may differ depending on the setting and we would appreciate some further clarification regarding your research. Could you please provide further details on why your study is exempt from the need for approval and confirmation from your institutional review board or research ethics committee (e.g., in the form of a letter or email correspondence) that ethics review was not necessary for this study? Please include a copy of the correspondence as an ""Other"" file.

The study is a technical signal processing benchmark and does not seek to extend biological or medical knowledge. Under French regulation (Loi Jardé), research that focuses on technical performance rather than clinical outcomes is not considered a clinical study involving the human person (RIPH) and is therefore exempt from formal Institutional Review Board (IRB) approval (Deplanque et al., 2017). This legal framework distinguishes between biomedical research and technical evaluations that do not impact patient care or medical theory.

To support this, we have attached an email correspondence from Mathilde Herbet, our referent Clinical Research Associate at the Clinical Investigation Center (CIC-IT). For your convenience, here is the English translation of the provided email:

*"I confirm that if the data collected for this research does not allow the person to be identified, directly or indirectly (no date, time, location, existing correspondence list), the GDPR does not apply because the data is completely anonymous. No declaration is necessary."*

3)We note that you have indicated that there are restrictions to data sharing for this study. PLOS only allows data to be available upon request if there are legal or ethical restrictions on sharing data publicly. For more information on unacceptable data access restrictions, please see http://journals.plos.org/plosone/s/data-availability#loc-unacceptable-data-access-restrictions.

Before we proceed with your manuscript, please address the following prompts:

a) If there are ethical or legal restrictions on sharing a de-identified data set, please explain them in detail (e.g., data contain potentially identifying or sensitive patient information, data are owned by a third-party organization, etc.) and who has imposed them (e.g., a Research Ethics Committee or Institutional Review Board, etc.). Please also provide contact information for a data access committee, ethics committee, or other institutional body to which data requests may be sent.

b) If there are no restrictions, please upload the minimal anonymized data set necessary to replicate your study findings to a stable, public repository and provide us with the relevant URLs, DOIs, or accession numbers. For a list of recommended repositories, please see

https://journals.plos.org/plosone/s/recommended-repositories. You also have the option of uploading the data as Supporting Information files, but we would recommend depositing data directly to a data repository if possible.

We will update your Data Availability statement on your behalf to reflect the information you provide.

In accordance with PLOS ONE’s data availability policy, we have provided the minimal anonymized data set necessary to replicate the study findings. This data set, including individual temporal and spectral characteristics, is uploaded as a **Supporting_Information_file.xlsx**. There are no further ethical or legal restrictions preventing the sharing of these de-identified data.

4) Please upload a new copy of Figures 1 to 9 as the detail is not clear. Please follow the link for more information: https://journals.plos.org/plosone/s/figures

We have updated the resolution of all figures to comply with the dpi requirement. All files have been successfully tested and validated using the PLOS NAAS tool. The resulting high-resolution TIFF files have been uploaded to the submission system.

**Response to Reviewer #1**

1) The paper largely confirms known physiological delays and frequency bands without offering new physiological insights, algorithmic advances, or clinically actionable thresholds. A simple descriptive study in 20 young healthy adults under resting conditions does not constitute a generalizable reference dataset.

We acknowledge that N=20 is a modest sample size for a clinical trial. However, we would like to clarify that this work is primarily a descriptive technical analysis aimed at optimizing signal processing frameworks rather than establishing universal clinical norms. Since the study was conducted on a homogeneous group of healthy volunteers, the inter-subject physiological variability remained low.

2) N=20 is small for establishing population wide benchmarks. No power calculation is provided. The study includes only healthy subjects, severely limiting generalizability to real world ambulatory monitoring.

We contend that the statistical unit of interest in this context is the cardiac waveform itself. Our analysis is based on several validated cardiac cycles per modality, providing high confidence in the stability of the reported morphological and spectral templates. We have updated the Discussion (Lines 334 – 342) to reflect this perspective and to acknowledge that while N=20 is sufficient for this technical benchmark, larger cohorts are required for broader clinical generalizability.

3) Aligning each cardiac cycle to the maximum amplitude within a predefined window artificially sharpens waveforms and reduces true temporal variability. This biases correlation and delay estimates. The median waveform computed after per cycle peak alignment is not a true ensemble average and cannot be directly compared with standard ECG averaging methods.

We acknowledge the reviewer's point that aligning waveforms on local maxima (within a ±20 ms window) may "sharpen" the resulting templates and potentially inflate Pearson’s correlation coefficients. However, we contend that this strategy is methodologically necessary for multimodal analysis.

The delay between the electrical activation (ECG R-peak) and the subsequent mechanical events (PCG, SCG, or PPG) is subject to physiological "jitter" or intra-individual variability, influenced by factors such as instantaneous heart rate, autonomic tone, and respiratory phase. Without this local alignment, the ensemble averaging process would act as a low-pass filter, blurring the rapid mechanical transients (like the S1 sound or SCG waves) and misrepresenting their true morphology.

By allowing for this fine-tuning, we compensate for these minor physiological fluctuations to capture the most representative "typical" waveform for each modality. We have added a comment in the Methods section (Lines 175 – 183) to clarify this trade-off between alignment precision and the resulting morphological consistency.

4) DC subtraction after windowing is appropriate, but the paper does not describe handling of baseline wander, motion artifacts, or outlier beat rejection.

We have refined the description of the signal preprocessing in the Methods section to provide more clarity. Baseline wander was handled using the 5 Hz high-pass component of our bandpass filter. Furthermore, we have detailed the outlier rejection used in our analysis, where beats with R-R intervals falling outside 2.5 standard deviations from the median were excluded (Lines 125 – 131).

5) At HR > 75 bpm, diastole shortens, yet the same PCG S2 window [+280, +500] is used for all subjects – a systematic error. PPG window [+150, +1350] corresponds to HR ≥ 45 bpm, but the study did not report actual HR ranges. No justification is given for choosing a 600 ms refractory period for R peak detection to favor specificity (this may discard real beats in subjects with resting HR near 100 bpm).

We agree that a static window would introduce systematic errors at high heart rates. However, the subjects' heart rates remained stable and low throughout the recordings, with a mean of 62.3 bpm (range: 57.1–71.4 bpm). We have updated the Results section to report these HR ranges (Lines 206– 207).

Regarding the 600 ms refractory period: as the maximum HR observed was 71.4 bpm (corresponding to an R-R interval of ~840 ms), this setting remained well below the actual physiological intervals, ensuring no real beats were discarded while maintaining high specificity against noise.

6) Power spectral density (PSD) is estimated on individual pulse windows (not long continuous segments). This is unusual and discards low frequency HRV information (<0.5 Hz).

We have clarified the PSD estimation strategy in the Methods section (Lines 175 – 183). The PSD was calculated on individual cardiac windows specifically to capture the spectral signature of the waveform morphology. By isolating the pulse from inter-beat intervals and continuous background noise, we ensure that the identified peak frequencies and bandwidths are directly related to the physiological "wave" of interest, providing better guidance for heartbeat detector optimization.

7) Intra vs. inter subject comparisons are presented without formal statistical testing.

We acknowledge the need for formal statistical validation to support our descriptive findings. We have now performed a Wilcoxon signed-rank test to compare intra-individual versus inter-individual Pearson correlation coefficients for each modality. We have updated Table 2 and Table 4 to include these p-values.

8) Pearson’s r is used to compare waveform morphology after amplitude normalization and temporal alignment to the peak. This inflates correlation.

We agree with the reviewer that synchronization and normalization mechanically increase the correlation coefficients. We have updated the Discussion section (Lines 334 - 342) to explicitly state that these preprocessing steps lead to an artificial inflation of Pearson’s compared to raw signals. We now clarify that while this approach is necessary to isolate and evaluate morphological consistency for detector design, it likely results in higher r values than would be observed without such alignment.

**Response to Reviewer #2**

1) The study is useful, but its novelty is mainly integrative rather than strongly methodological. The main contribution is the unified comparison of several heartbeat sensing modalities under one system, rather than a new sensing principle, algorithm, or physiological model. That positioning is acceptable, but the manuscript should state it more clearly and avoid overstating its general significance.

We have updated the Introduction (Lines 49 – 55) to more clearly define the study's scope and novelty. We now emphasize the integrative nature of our work, highlighting that the primary contribution is the unified comparison of five modalities recorded simultaneously under identical conditions. This clarifies that the study serves as a technical benchmark for multisensor fusion and standardized signal processing in healthy populations.

2) The alignment strategy may improve visual consistency, but it may also bias waveform similarity and delay interpretation. The manuscript states that signal-specific waves were extracted around local maxima within predefined windows, and that delays were artificially nullified for visualization. The authors also acknowledge that this may artificially sharpen the resulting waveform, especially for more variable signals such as PCG and SCG. This issue should be more carefully discussed, because some of the reported consistency may partly come from the processing strategy itself.

We agree with the reviewer that synchronization and normalization mechanically increase the correlation coefficients. We have updated the Discussion section (Lines 343 - 349) to explicitly state that these preprocessing steps lead to an artificial inflation of Pearson’s compared to raw signals. We now clarify that while this approach is necessary to isolate and evaluate morphological consistency for detector design, it likely results in higher r values than would be observed without such alignment.

3) The conclusions should be more cautious because the results are system-dependent and based on a limited experimental setting. The frequency-domain findings are influenced by hardware response, and this is particularly relevant because the PCG sensor description and the reported dominant PCG frequency range raise questions about how much of the spectral result reflects physiology versus instrumentation. In addition, the study only includes 20 healthy adults under resting conditions, and the statistical analysis is mainly descriptive. Therefore, the conclusions should be framed more explicitly as baseline observations under controlled conditions, rather than broadly generalizable modality-level claims.

We agree that the hardware response is a critical factor in spectral analysis. We have added the following technical information to the manuscript to address this:

- SCG: We added (Lines 75 – 76) the specific bandwidth of the ADXL356 (2400 Hz on the Z-axis) to the material description to show that the sensor did not limit the captured vibrations.
- PCG: We added (Lines 72-73) the bandwidth and noise performance of the TS472 preamplifier to the material description to confirm high-fidelity acoustic capture.
- Sampling Rate: We explicitly addressed (Lines 87 – 90) the 1000 Hz sampling rate limitation in the Methods and Discussion sections, clarifying that while it may limit high-frequency harmonic analysis, it is sufficient for the study's primary goal of heart rate detection optimization.
- Added Wilcoxon signed-rank tests (p-values) to Tables 2 and 4 to formally compare intra- vs. inter-subject correlations. This demonstrates that despite the small N=20, the observed morphological patterns are statistically significant.

We have clarified in the Discussion (Lines 301 – 307, 317 – 320 & 324 - 327) that these benchmarks are system-dependent and provide a reference for integrated monitoring systems using similar noninvasive technologies.

**Response to Reviewer #3**

1) The study only includes 20 healthy adult volunteers aged 24 to 52. To make the proposed signal processing guidelines truly robust, the authors should discuss how these time’s frequency characteristics might shift in older populations or patients with cardiovascular pathologies.

We have updated the discussion section (Lines 334 – 342) to discuss population diversity and physiological factors. We explicitly acknowledge that BMI, which was not recorded, likely influences the attenuation of mechanical vibrations (SCG/PCG) through adipose tissue. We also discussed how age and cardiovascular pathologies would shift the observed benchmarks due to changes in arterial stiffness and cardiac mechanics.

2) A fixed refractory period of 600 ms was used for ECG R-peak detection. While appropriate for resting conditions, the authors should address how this algorithm could be dynamically adapted for ambulatory or exercise monitoring where HR frequently exceeds 100 bpm.

We agree that a fixed 600 ms refractory period is a limitation for high heart rate monitoring. However, in the context of this resting-state study, the heart rates remained between 57.1 and 71.4 bpm (mean: 62.3 ± 4.2 bpm).

With a maximum observed heart rate of 71.4 bpm, the minimum R-R interval was approximately 840 ms, leaving a safety margin of 240 ms before the refractory period would interfere with true QRS complexes. We have added this observation to the Discussion (Lines 350 – 353) to clarify the algorithm's scope.

3) The extraction windows for PCG and SCG are defined using fixed ranges. Given that systolic and diastolic durations vary non linearly with heart rate, relying on static time windows relative to the R-peak might cause truncation or overlap at higher heart rates. This limitation should be explicitly discussed.

The reviewer raises a valid physiological point regarding the non-linear shortening of the cardiac cycle phases (especially diastole) as heart rate increases. In our study, the use of static windows was justified by the stability of the resting heart rates (mean 62.3 bpm). We have updated the Results section to report these HR ranges (Lines 206 – 207) and address this point in discussion (Lines 345 – 359).

4) The ECG is bandpass filtered at 5-15 Hz before peak detection. However, the paper later states the peak frequency for QRS is 11 Hz with a bandwidth up to 18 Hz. The authors should confirm that the 15 Hz low-pass cutoff did not artificially attenuate the QRS complex or induce phase shifts affecting the exact latency measurements.

The 5–15 Hz bandpass filter was used exclusively for robust R-peak detection (Pan & Tompkins type) to maximize specificity. All quantitative analyses were performed on the raw, unfiltered signal. This approach ensures that no phase shift or QRS attenuation affected our measurements. We added a clarification in the *Methods* section (Lines 128 – 131) of the manuscript to explicitly distinguish the filtered detection stage from the raw data analysis stage.

5) Table 1 shows substantial inter-subject variability for SCG. The authors attribute this to "sensor coupling and anatomical factors", but a more detailed analysis on whether body mass index or gender influenced this mechanical signal attenuation would add significant clinical value.

We acknowledge that these anthropometric factors were not recorded in the current study, which is a limitation. Physiologically, variations in chest wall thickness and adipose tissue (related to BMI and gender) are known to act as a low-pass filter for mechanical vibrations, explaining part of the inter-subject differences in waveform morphology and amplitude seen in Table 1. We have updated the Discussion section (Lines 334 – 342) to explicitly address these factors as contributors to the observed variability and as essential parameters for future clinical standardizations.

6) The spectral peaks for PCG were both found to be around 30~31 Hz. Physiological literature often notes differences in frequency content between the mitral or tricuspid closure and pulmonary closure. The authors should comment on why their analysis did not capture this expected distinction.

While physiological literature often notes spectral differences between heart sounds, our study identifies the primary power peak, which remained centered around 30–31 Hz. This similarity likely results from the resting state of our subjects, where fundamental frequency components tend to overlap (Moukadem et al., 2015), and our specific recording site at the pulmonary area which emphasizes the lower-frequency P2 component. Furthermore, while the total energy envelope may extend to higher ranges, the dominant power density reflects the fundamental mechanical resonance of the cardio-hemic system (Brusco & Nazeran, 2004; Narváez et al., 2020). We have added these precisions to the Discussion section to clarify why this expected distinction was not captured (Lines 301 – 307).

7) A sampling rate of 1000 Hz is used for all signals. While sufficient for ECG and PPG, PCG components can extend up to several hundred Hertz. The authors should clearly state that 1000 Hz might limit the analysis of higher acoustic harmonics.

We acknowledge that a 1000 Hz sampling rate limits the frequency analysis to a 500 Hz Nyquist frequency, which may underrepresent higher acoustic harmonics or rapid murmurs in the PCG signal. However, for the specific objective of this study—heartbeat detection and multimodal synchronization—this bandwidth is more than sufficient.

As established in the literature, the primary energy and diagnostic features of the S1 and S2 heart sounds are concentrated below 150 Hz. By focusing on this core spectral range, we ensure a robust characterization of the temporal benchmarks across all subjects. We have updated the Methods (Lines 87 – 90) and Discussion (Lines 324 – 327) sections to explicitly state this trade-off, clarifying that while fine acoustic harmonics are not the focus, the 1000 Hz rate is optimal for characterizing the dominant energy of integrated cardiac sensors.

8) The PPG wave peaks around 378 ms post R-peak. The authors should mention whether their evaluation considered the subjects' heights or arm lengths, as these anatomical dimensions directly dictate the physical Pulse Transit Time delay to the fingertip.

The reviewer is correct that anatomical dimensions, such as height and arm length, directly influence the Pulse Transit Time (PTT) to the fingertip. In this study, we did not record or use these individual measurements to normalize the temporal delays for PPG and PiPG.

Consequently, the reported delays reflect the raw physiological variability inherent in a diverse adult population. We have added a dedicated paragraph in the Discussion (Lines 360 – 365) to address this: we clarify that this lack of anatomical normalization contributes to the inter-subject variability observed in Table 3. This highlights the importance of considering individual morphology when defining absolute physiological constants in clinical applications.

9) Data was collected under resting conditions to minimize artifacts. A discussion on how typical motion artifacts specifically affect the distinct frequency bands of PiPG vs. PPG is recommended.

To address the reviewer's comment on motion artifacts, we have added a concise comparison in the Discussion section (Lines 329 – 331). We clarify that PPG is mostly affected by low-frequency baseline fluctuations, while PiPG is sensitive to a broader spectrum of mechanical perturbations due to its piezoelectric nature. This addition highlights the distinct signal-processing challenges for each modality in non-resting conditions.

Sincerely,

Pierre Charlier, PhD

Corresponding Author

CHU Lille, France
