# Supplementary material for: Time and frequency characteristics of various noninvasive heartbeat sensors
Source: PLoS One. 2026 Sep 3;21(9):e0357922. doi: 10.1371/journal.pone.0357922 (PMC13541146; doi:10.1371/journal.pone.0357922)
Supplement: Supplementary file 2 [file pone.0357922.s004.docx]

| Pierre Charlier  CHU Lille, CIC 1403  Lille, France  [pierre1.charlier@chu-lille.fr](mailto:pierre1.charlier@chu-lille.fr) | July 17, 2026 |
| --- | --- |

Subject: Submission PONE-D-26-10913

Dear Dr. Ming Zhang and Reviewers,

Thank you for the opportunity to submit a revised version of our manuscript, "Time and Frequency Characteristics of various noninvasive heart beat Sensors". We appreciate the time and effort the reviewers dedicated to providing insightful feedback. We have addressed all concerns, which have significantly strengthened our paper.

Below is our point-by-point response to the reviewers' comments.

**Reviewer #1 and Reviewer #2**

We thank both reviewers for their positive evaluation and for approving the manuscript.

**Reviewer #3**

1) The simultaneous acquisition of five cardiac signal modalities is a strength. However, the manuscript should clarify whether its main contribution is a physiological comparison, a hardware-validation study, a signal-processing benchmark, or a publicly reusable reference dataset.

We agree with the reviewer and have revised the last paragraph of the Introduction to clarify our main contribution. We explicitly state that this work aims to provide a "signal-processing benchmark and physiological comparison" rather than a "hardware-validation study".

2) The sample is limited to 20 healthy adults recorded for five minutes under resting conditions. No sample-size justification is provided. Claims regarding general modality characteristics, wearable monitoring, and standardization should therefore be restricted to this small, healthy, resting cohort.

We agree with this observation. We have updated the Conclusion section to explicitly restrict our claims regarding general modality characteristics and standardization to the specific cohort studied (a small cohort of healthy adults under resting conditions).

3) The reported spectra may reflect the specific sensors and analog front-end rather than the physiological modalities themselves. Full frequency-response curves, gains, hardware filters, anti-aliasing procedures, calibration information, and noise floors should be reported for every acquisition channel.

We have updated the *Methods* section to explicitly report these hardware parameters based on the analog front-end specifications. We added details specifying its high linearity, the input-referred noise floor and clarified that anti-aliasing is managed by the device.

4) The microphone is described as having a stable response from 50 Hz, yet the reported dominant PCG frequencies are approximately 30–31 Hz. The authors must demonstrate that these low-frequency measurements are reliable after considering the complete microphone–preamplifier–ADC transfer function.

We thank the reviewer for this important comment. We acknowledge that the manufacturer specifies the microphone operating frequency range from 50 Hz to 16 kHz and does not provide a calibrated frequency response below 50 Hz. We therefore cannot claim a fully characterized response in this frequency range. However, we performed internal measurements on the complete acquisition chain (microphone, preamplifier, and ADC), which demonstrated that the system remains sensitive to frequencies down to approximately 15 Hz, although the response is no longer perfectly linear. Consequently, the reported spectral components around 30 Hz should be interpreted as indicative of the dominant frequency content rather than as absolute calibrated amplitude measurements. We have clarified this limitation in the revised manuscript.

5) The alignment procedure may artificially increase waveform similarity. Individual signals are centered on local maxima within predefined windows, and the manuscript acknowledges that this may sharpen median waveforms. Results should be compared with an ECG-only alignment analysis to quantify the bias introduced by modality-specific re-centering.

We thank the reviewer for this remark. While an ECG-only alignment is a standard approach, it is physiologically suboptimal for this study. As shown by our results in Table 3, we observed significant intra-individual timing jitter (standard deviations ranging from ±17.53 ms for PiPG to ±48.24 ms for PPG). Relying on a fixed ECG-only alignment over 5-minute recordings would result in a severe low-pass filtering effect, which would completely deform the median waveform morphology and obscure rapid mechanical transients. We maintain that the local maximum recentering is a necessary procedure to compensate for this natural physiological variability and to preserve the true morphology of the signals; justification is present in the materials & methods section.

6) Inter-subject similarity appears to compare each participant with a grand median that includes that participant’s own waveform. This creates circularity and may inflate correlations. A leave-one-subject-out reference waveform should be used for each participant.

We thank the reviewer for this observation. We agree that the use of a grand median including the subject under evaluation may introduce a small degree of circularity and potentially overestimate inter-subject similarity. To address this concern, the inter-subject analysis was revised using a leave-one-subject-out approach. For each participant, the reference waveform was recomputed from all remaining participants, excluding the waveform of the subject being evaluated. The corresponding correlation values were recalculated and the manuscript has been updated accordingly.

7) Pearson correlation alone is insufficient for assessing reproducibility because it is affected by alignment and is insensitive to amplitude differences after normalization. The authors should supplement it with confidence intervals and appropriate measures of timing, morphology, and agreement, such as latency variability, normalized error, dynamic time warping, or intraclass correlation.

We thank the reviewer for this valuable comment. We agree that additional agreement metrics such as latency variability, normalized error, dynamic time warping, or intraclass correlation can provide complementary information depending on the study objective. In the present work, however, our primary objective was to provide a descriptive characterization of the reproducibility of the waveform morphology for signal processing applications rather than to perform a comprehensive method-comparison study. Since all signals were preprocessed and amplitude-normalized prior to analysis, absolute amplitude differences were not considered relevant, as they are highly dependent on the sensing modality, sensor characteristics, and placement rather than on the underlying physiological event. For this reason, Pearson correlation was selected to quantify waveform reproducibility after normalization. We acknowledge that additional agreement metrics could further complement the analysis and will consider including them in future work.

8) Welch PSD is applied to relatively short event windows using a 2048-point FFT, which likely involves extensive zero-padding. The authors should report the actual segment duration, effective frequency resolution, detrending and windowing procedures, and sensitivity of peak-frequency estimates to analysis settings.

We thank the reviewer for this insightful comment. We have clarified the spectral analysis methodology in the revised manuscript by specifying the segment duration, frequency resolution, detrending procedure, windowing parameters, and FFT settings. Specifically, the Welch PSD analysis was performed on isolated cardiac peak windows with durations ranging from 200 ms to 400 ms, resulting in an intrinsic frequency resolution of 2.5 to 5 Hz. Each segment was detrended by subtracting its mean value to remove DC bias, and a Hamming window with 50% overlap was applied using the MATLAB pwelch function. A 2048-point FFT was used to provide finer spectral interpolation of the frequency domain without affecting the intrinsic frequency resolution. These details have been added to the revised Methods section to improve the reproducibility and clarity of the spectral analysis.

9) Since the study emphasizes multimodal sensing and signal integration, the authors may briefly discuss broader advances in multimodal integrated sensing systems. For reference, see DOI: 10.1109/MCOM.004.2400281. Such discussion should remain secondary to directly relevant biomedical sensor-fusion literature.

This publication aims to provide a descriptive analysis of various cardiac signal acquisition technologies in order to guide designers and developers in their technological choices regarding signal acquisition and processing techniques. This work therefore emphasizes sensors technologies description, rather than multimodal sensing which, in our view, constitutes a separate research topic.

10) The physiological interpretation of the spectral peaks should be more cautious. For PPG and PiPG, the dominant 1–2 Hz component may largely reflect the participant’s heart rate rather than an intrinsic modality-specific bandwidth. Analyses separating the fundamental cardiac frequency from waveform harmonics would be more informative.

We acknowledge the reviewer's point regarding the interpretation of spectral peaks. We have revised our discussion to clarify that for PPG and PiPG, the 1–2 Hz dominant component is essentially governed by the fundamental heart rate. However, we emphasize that since these signals are hemodynamic pulse waves, their usable bandwidth is physiologically tied to the cardiac cycle duration. We have added a statement in the Discussion specifying that for these modalities, the spectral content is inherently linked to the patient's heart rate, which justifies this frequency range as the primary observation window.

11) SCG1 and SCG2 are defined by their temporal correspondence with PCG windows rather than established SCG fiducial events. Without an independent mechanical or imaging reference, the manuscript should avoid implying that these peaks directly identify specific valve events.

We completely agree. We have carefully reviewed the manuscript and removed any ambiguous phrasing that could imply these SCG peaks directly identify specific mechanical valve events. We ensured they are strictly defined by their temporal correspondence with the PCG windows.

We have reformulated the relevant section in the Methods to explicitly state that the terms SCG1 and SCG2 are used strictly based on their temporal correspondence with PCG windows, without implying that these components directly identify specific valve events.

12) The statistical analysis is mostly descriptive, yet terms such as significant similarity are used. The authors should report confidence intervals, participant-level distributions, paired comparisons across modalities, and sensitivity analyses. The numbers of excluded beats, failed detections, motion artifacts, and manually corrected peaks should also be disclosed.

We thank the reviewer for this comment. The objective of this study was primarily descriptive and focused on the characterization of the extracted physiological features and their similarity across sensing modalities under controlled resting conditions. Therefore, the statistical analysis was designed to provide descriptive metrics of waveform morphology and spectral consistency rather than to perform hypothesis-driven comparisons between modalities.

To improve transparency, we have added the number of excluded cardiac cycles resulting from the automated RR outlier rejection procedure. Across the 20 recordings, 6360 cardiac cycles were detected, and 171 cycles (2.7%) were excluded based on the predefined RR interval rejection criterion. No manual peak corrections were performed. Motion artifacts were not specifically quantified because the recordings were performed under controlled conditions, with all subjects resting during acquisition, resulting in minimal motion-related disturbances.

Sincerely,

Pierre Charlier, PhD

Corresponding Author

CHU Lille, France
