# Supplementary material for: Time and frequency characteristics of various noninvasive heartbeat sensors
Source: PLoS One. 2026 Sep 3;21(9):e0357922. doi: 10.1371/journal.pone.0357922 (PMC13541146; doi:10.1371/journal.pone.0357922)
Supplement: Supplementary file 3 [file pone.0357922.s005.docx]

| Pierre Charlier  CHU Lille, CIC 1403  Lille, France  [pierre1.charlier@chu-lille.fr](mailto:pierre1.charlier@chu-lille.fr) | August 17, 2026 |
| --- | --- |

Subject: Submission PONE-D-26-10913

Dear Dr. Ming Zhang and Reviewers,

Below is our point-by-point response to the reviewers' comments.

**Reviewer #3**

1) The PCG frequency concern is only partially addressed. The authors state that internal testing showed sensitivity down to approximately 15 Hz, but no calibration protocol, transfer-function curve, reference source, uncertainty, or raw measurement is provided. The reported 30 Hz peak should remain explicitly device-specific and qualitative.

We fully agree with the reviewer's assessment. We have updated the Discussion section (methodological limitations) to explicitly state that because no formal calibration protocol or transfer-function curve was established below 50 Hz for this specific setup, the reported 30 Hz peak for the PCG signal must be considered explicitly device-specific and qualitative.

2) The requested alignment-control analysis was not performed. Instead, the authors justify local peak recentering while also acknowledging that normalization and alignment mechanically inflate correlations. A direct ECG-locked versus locally aligned comparison is still needed to quantify this effect.

We apologize for not including this quantitative comparison in the previous revision. We have now performed a strict ECG-locked alignment analysis without local peak recentering. As expected, without compensating for physiological timing jitter, the strict ECG-locked ensemble averaging mechanically reduced the correlation coefficients by an average of delta r = -0.47 for PCG and delta r = -0.50 for SCG (detailed comparison provided in **Supporting Information**). These quantified results have also been integrated into the *Results* and *Methods* sections of the revised manuscript. This confirms that while local re-centering inflates raw waveform similarity, it remains a necessary step to compensate for the flattening effect of physiological timing jitter and preserve the true morphology of the signals.

3) The leave-one-subject-out reference analysis appropriately resolves the previous circularity concern. This is an important improvement to the inter-subject comparison.

We thank the reviewer for this positive feedback and for validating this methodological improvement.

4) The reproducibility analysis remains incomplete. The authors retain Pearson correlation alone and provide mean ± standard deviation, but no confidence intervals or complementary morphology/agreement metrics. Describing high correlations as strong physiological reproducibility is therefore still too broad.

We acknowledge this limitation. We have updated Tables 2 and 4 to include the 95% Confidence Intervals (CI) for all Pearson correlation coefficients, calculated using the Fisher z-transformation. Finally, we have toned down our claims in the Discussion, replacing broad statements like "strong physiological reproducibility" with more measured terms such as "high morphological agreement."

5) Since the manuscript continues to emphasize multimodal integration and future sensor-fusion applications, a brief discussion of related multimodal sensing developments remains relevant. For reference, see DOI: 10.1109/MCOM.004.2400281. This should remain secondary to biomedical literature.

We have added a brief statement to the end of the Introduction acknowledging broader developments in multimodal integrated sensing systems, and we have incorporated the suggested reference (DOI: 10.1109/MCOM.004.2400281), while ensuring it remains secondary to the core biomedical literature.

Sincerely,

Pierre Charlier, PhD

Corresponding Author
