# Supplementary material for: Time and frequency characteristics of various noninvasive heartbeat sensors
Source: PLoS One. 2026 Sep 3;21(9):e0357922. doi: 10.1371/journal.pone.0357922 (PMC13541146; doi:10.1371/journal.pone.0357922)
Supplement: Supplementary file 4 [file pone.0357922.s006.docx]

| Pierre Charlier  CHU Lille, CIC 1403  Lille, France  [pierre1.charlier@chu-lille.fr](mailto:pierre1.charlier@chu-lille.fr) | August 21, 2026 |
| --- | --- |

Subject: Submission PONE-D-26-10913

Dear Dr. Ming Zhang and Reviewers,

Below is our point-by-point response to the reviewers' comments.

**Reviewer #3**

1) The manuscript would benefit from a careful overall formatting revision. Please check the consistency of capitalization in the title and section headings, as well as font style, line spacing, paragraph indentation, and spacing before and after headings throughout the manuscript.

We agree with the reviewer's assessment. We have standardized the font style and paragraph indentation throughout the text. Furthermore, we have verified the consistency of capitalization in the title and all section headings, and ensured uniform spacing before and after headings to comply with standard academic formatting.

2) The formatting of figures and tables should be further standardized. Please ensure that all figure/table captions follow a consistent style, that abbreviations and units are presented uniformly, and that the numbering and in-text citations of figures and tables are carefully checked.

We have carefully reviewed and standardized all figures and tables. Figure and table captions now follow a strictly uniform style (e.g., standardizing the "Fig X." prefix). We have also ensured that all abbreviations (such as wave components) and units (adhering to SI conventions) are presented uniformly across all visual elements and the main text. Finally, we verified the sequential numbering and corrected the in-text citations to ensure proper referencing throughout the manuscript.

3) The reference and citation formatting should be carefully revised. Please ensure that all references follow a consistent journal style, including author names, journal titles, volume/issue information, page ranges, DOI presentation, and in-text citation format. The authors are also encouraged to expand the reference list to approximately 30 relevant references, particularly by including more recent studies related to noninvasive cardiac sensing, wearable monitoring, multimodal physiological signals, and sensor fusion.

We have carefully revised the reference and citation formatting. To ensure strict compliance and consistency, we have completely rebuilt our bibliography using the Zotero reference management software, applying the Vancouver style as required by the journal. Furthermore, the bibliography has been updated and expanded to 30 references to include recent studies related to noninvasive cardiac sensing, wearable monitoring, and sensor fusion, as suggested by the reviewer.

4) Recent studies in DOI: (10.1016/j.sigpro.2026.110584), (10.1016/j.measurement.2025.118915), (10.1109/SMC58881.2025.11342493), (10.1016/j.bspc.2026.110367), and (10.1016/j.neucom.2025.130151) provide relevant background for this work. Including these studies would strengthen the positioning and novelty discussion of this manuscript.

We thank the reviewer for the suggested references regarding advanced signal processing and feature extraction. We have carefully evaluated them and integrated the most relevant regarding multi-feature fusion and robust signal processing architectures into our Introduction. Furthermore, we have expanded our bibliography with recent studies directly focused on multimodal cardiac monitoring, sensor fusion (ECG, SCG, PPG, and PiPG), and wearable physiological acquisition.

5) The overall presentation of the manuscript still requires careful formatting and proofreading. There are many inconsistencies in capitalization, hyphenation, spacing, and terminology throughout the manuscript. For example, the title uses inconsistent capitalization (“various”, “Sensors”), and “heart beat” would normally be written as “heartbeat”. Terms such as “R peak/R-peak”, “time domain/time-domain”, “inter-subject/intersubject”, and “intra-individual/intra individual” should also be standardized throughout the manuscript.

We apologize for these editorial oversights. We have thoroughly proofread the manuscript to resolve all formatting, typesetting, and terminology inconsistencies.

6) The formatting of physical quantities and units should be thoroughly standardized according to SI conventions. A space should be inserted between numerical values and units, e.g., “600 ms” instead of “600ms”, “20 ms” instead of “20ms”, and “11 Hz” instead of “11Hz”. Similar inconsistencies occur repeatedly in the Methods, Results, and Discussion. The notation “100 min-1” should preferably be typeset as “100 min⁻¹” or expressed consistently as bpm.

We have carefully reviewed the manuscript and standardized the formatting of physical quantities according to SI conventions. Spaces have now been systematically inserted between numerical values and their respective units (e.g., "600 ms", "11 Hz"). Furthermore, heart rate units are now consistently expressed as "bpm" throughout the text.

7) Please carefully verify the units associated with sensor specifications. For example, the SCG accelerometer noise density is reported as “80 μg/Hz”. Since noise density is commonly expressed with a square-root bandwidth term, the authors should verify whether the intended unit is μg/√Hz and correct it if necessary.

We thank the reviewer for spotting this typographical error. We have corrected the noise density unit to "80 μg/√Hz" in the *Methods* section. We also proactively corrected a similar unit error for the PCG preamplifier (10 nV/√Hz).

8) The figure quality and visual consistency need substantial improvement. Several figures contain very small labels, thin lines, low-contrast elements, and excessive blank space. This is particularly evident in Figs. 5–9. For example, the axes and labels in Fig. 5 are difficult to read, while Figs. 8 and 9 contain multiple stacked panels with relatively small fonts. All figures should use consistent font sizes, line widths, axis styles, and labeling conventions and should remain readable at the final journal column width.

We thank the reviewer for this valuable feedback. We have thoroughly revised and improved Figures 5 through 9 to enhance visual clarity and consistency. Specifically, we increased the font sizes for all axis labels and numbers, thickened the line widths, and adjusted the layouts to eliminate excessive blank space.

9) The authors should avoid relying primarily on color to distinguish important graphical information. In Figs. 8 and 9, individual curves and median curves are mainly differentiated using grey and red. Please consider combining color with different line styles/line widths so that the figures remain interpretable in grayscale printing and for readers with color-vision deficiency. High-resolution or preferably vector-format graphics should also be provided.

We have updated Figures 8 and 9 by combining color differentiation with distinct line widths and line styles. Furthermore, improved-resolution graphics have been provided for all figures

10) The arrangement of figure files in the submitted reviewer PDF should be checked. Figure 9 appears before Figures 1–8 in the appended figure section. Even if the figures are uploaded separately during submission, the final reviewer file should ideally present them in numerical order to facilitate reading and cross-checking.

We thank the reviewer for pointing this out. We will ensure that all figure files are uploaded in correct numerical order during the submission process.

11) Figure captions should be standardized and made more self-contained. There are inconsistent styles such as “Fig 1:”, “Fig 7 :”, and similar spacing differences before colons. Captions should use one journal-compliant format consistently. In addition, captions for multi-panel or information-dense figures should explain the curves, colors, shaded regions, abbreviations, and axes sufficiently without requiring extensive consultation of the main text.

We have standardized all figure captions to follow a consistent, journal-compliant format (e.g., "Fig 1."). Furthermore, we have expanded the captions for multi-panel and information-dense figures (specifically Figs 8 and 9) to fully explain the curves, colors, and reference axes, ensuring they are entirely self-contained without needing to consult the main text.

12) The tables would benefit from a more professional journal-style layout. Tables 1–5 currently use dense full gridlines and relatively compact formatting. The authors may consider a cleaner table design with fewer horizontal/vertical rules, consistent decimal alignment, units placed in column headings, and uniform footnote notation. In addition, terms such as “Broadband (-3 dB)” in Table 5 should be reconsidered; “−3 dB bandwidth” would be more technically conventional and consistent with the Methods section.

We have completely reformatted Tables 1–5 to adhere to a professional, journal-compliant layout. We removed all vertical rules, minimized horizontal rules, aligned decimals, and standardized footnote notations. Additionally, the term “Broadband (-3 dB)” has been updated to “−3 dB bandwidth” in Table 5 to ensure consistency with the *Methods* section.

13) There are apparent inconsistencies between numerical values reported in the text and those presented in the tables, which should be carefully checked. For example, the Results text reports ECG, PPG, and PiPG correlations of approximately 0.91, 0.92, and 0.96, whereas Table 2 reports corresponding inter-individual values of 0.90, 0.90, and 0.96. Similar discrepancies appear in the spectral-domain results, where the text reports PCG S1/S2 values around 0.79/0.84 while Table 4 shows 0.78/0.83. These differences suggest that some parts of the text or tables may not have been fully synchronized after revision.

We deeply apologize for these discrepancies, which resulted from an incomplete text update following our latest data reprocessing. We have carefully cross-checked all numerical values in the text against the tables. The manuscript text has been fully synchronized with the correct, definitive values presented in Tables 2 and 4.

14) Statistical reporting should be made more precise and consistent. Tables 2 and 4 report essentially all comparisons simply as “p < 0.05”. If available, exact p-values would provide more informative statistical reporting. The authors should also clarify whether any correction for multiple comparisons was considered, given that several modalities are evaluated simultaneously. The formatting of confidence intervals should also be standardized, for example using en dashes consistently rather than mixed hyphen styles.

We have updated Tables 2 and 4 to report exact p-values as $p < 0.001$. As this is a descriptive study evaluating each modality independently against its reference, no multiplicity correction was applied, which is now explicitly stated in the Statistical analysis section. Finally, all confidence intervals in Tables 2 and 4 have been standardized to use en dashes.

15) A careful language and typesetting check is still needed. Several obvious editorial issues remain, such as “reference.Then” without a space, “fingers Sensors positioning”, inconsistent capitalization of “Human Beings”, and phrases such as “presented Table 1”, “As illustrated Fig 6”, or “summarized Table 2”, where “in” is missing. These issues do not affect the scientific meaning but significantly reduce the professional appearance of the manuscript.

We apologize for these editorial oversights. We have thoroughly proofread the manuscript and corrected these typographical errors, including capitalization (e.g., "human beings"), missing spaces, and the addition of missing prepositions ("in") before figures and tables.

16) The reference section requires urgent verification. The manuscript cites references with numbers well beyond 8, including citations such as (12), (18), (19), (20), (23), (24), and (25), whereas the reference list visible in the submitted PDF appears to end at Reference 8 before the figure files begin. The authors should confirm that all cited references are included and that citation numbering is correct throughout the manuscript.

We sincerely apologize for this oversight. The truncation of the reference list in the submitted PDF occurred because we had not updated and synchronized the final bibliography list with our Zotero reference manager after adding several in-text citations. We have now fully updated the bibliography using Zotero, ensuring that all cited references are properly included and that the citation numbering is strictly correct and sequential throughout the manuscript.

17) The reference formatting itself should also be standardized. Some entries contain “[Internet]”, access dates, “Available from:” URLs, and DOI information, whereas other references contain only DOI information. Please carefully check all references against the journal style and ensure consistent formatting of journal abbreviations, dates, page ranges, URLs, and DOIs.

Using Zotero, we applied a consistent journal citation style to ensure uniform formatting across all entries, specifically removing redundant '[Internet]' tags, access dates, and 'Available from' URLs.

18) The manuscript would benefit from a final consistency check of terminology used in figures, tables, and the main text. For example, “PCGS1/PCGS2”, “PCG (S1)/(S2)”, “SCG1/SCG2”, and “SCG (SCG1)/(SCG2)” are used in different locations. A single notation scheme should be adopted throughout all figures, tables, captions, and textual descriptions.

We agree that the terminology was inconsistent. We have now adopted a single, uniform notation scheme throughout the manuscript, figures, and tables: "PCG (S1)", "PCG (S2)", "SCG (SCG1)", and "SCG (SCG2)".

19) The manuscript should also ensure that all mandatory reporting statements are presented clearly and in journal-compliant form. In particular, the ethics/waiver statement should clearly identify the responsible authority or regulatory basis, and the availability of the underlying dataset should be explicitly stated, especially because the Abstract describes the outcome as a “reference dataset”.

We have updated the mandatory reporting statements to fully comply with journal guidelines. The ethics statement in the *Methods* section has been revised to explicitly cite the regulatory basis for the waiver under French law (Loi Jardé). Furthermore, an explicit "Data Availability Statement" has been added to the manuscript, clarifying that while aggregated data and additional analyses are included in the Supporting Information files, the underlying raw reference dataset is available from the corresponding author upon reasonable request due to institutional data protection policies.

Sincerely,

Pierre Charlier, PhD

Corresponding Author
