# Supplementary material for: Time and frequency characteristics of various noninvasive heartbeat sensors
Source: PLoS One. 2026 Sep 3;21(9):e0357922. doi: 10.1371/journal.pone.0357922 (PMC13541146; doi:10.1371/journal.pone.0357922)
Supplement: Peer review history

# Peer review: Decision Letter 0

**DOI:** 10.1371/journal.pone.0357922.r001

**Authors:** Ming Zhang

20 Apr 2026

Dear Dr. CHARLIER,

Thank you for submitting your manuscript to PLOS ONE. After careful consideration, we feel that it has merit but does not fully meet PLOS ONE’s publication criteria as it currently stands. Therefore, we invite you to submit a revised version of the manuscript that addresses the points raised during the review process.

Please submit your revised manuscript by Jun 04 2026 11:59PM. If you will need more time than this to complete your revisions, please reply to this message or contact the journal office at plosone@plos.org. When you're ready to submit your revision, log on to https://www.editorialmanager.com/pone/ and select the 'Submissions Needing Revision' folder to locate your manuscript file.

- A letter that responds to each point raised by the academic editor and reviewer(s). You should upload this letter as a separate file labeled 'Response to Reviewers'.
- A marked-up copy of your manuscript that highlights changes made to the original version. You should upload this as a separate file labeled 'Revised Manuscript with Track Changes'.
- An unmarked version of your revised paper without tracked changes. You should upload this as a separate file labeled 'Manuscript'.

If you would like to make changes to your financial disclosure, please include your updated statement in your cover letter. Guidelines for resubmitting your figure files are available below the reviewer comments at the end of this letter.

If applicable, we recommend that you deposit your laboratory protocols in protocols.io to enhance the reproducibility of your results. Protocols.io assigns your protocol its own identifier (DOI) so that it can be cited independently in the future. For instructions see: https://journals.plos.org/plosone/s/submission-guidelines#loc-laboratory-protocols. Additionally, PLOS ONE offers an option for publishing peer-reviewed Lab Protocol articles, which describe protocols hosted on protocols.io. Read more information on sharing protocols at https://plos.org/protocols?utm_medium=editorial-email&utm_source=authorletters&utm_campaign=protocols.

As the corresponding author, your ORCID iD is verified in the submission system and will appear in the published article. PLOS supports the use of ORCID, and we encourage all coauthors to register for an ORCID iD and use it as well. Please encourage your coauthors to verify their ORCID iD within the submission system before final acceptance, as unverified ORCID iDs will not appear in the published article. *Only*  the individual author can complete the verification step; PLOS staff *cannot*  verify ORCID iDs on behalf of authors.

We look forward to receiving your revised manuscript.

Kind regards,

Ming Zhang

Academic Editor

PLOS One

**Journal Requirements:**

When submitting your revision, we need you to address these additional requirements.

1. Please ensure that your manuscript meets PLOS ONE's style requirements, including those for file naming. The PLOS ONE style templates can be found at

https://journals.plos.org/plosone/s/file?id=wjVg/PLOSOne_formatting_sample_main_body.pdf and

https://journals.plos.org/plosone/s/file?id=ba62/PLOSOne_formatting_sample_title_authors_affiliations.pdf

2. You indicated that ethical approval was not necessary for your study. We understand that the framework for ethical oversight requirements for studies of this type may differ depending on the setting and we would appreciate some further clarification regarding your research. Could you please provide further details on why your study is exempt from the need for approval and confirmation from your institutional review board or research ethics committee (e.g., in the form of a letter or email correspondence) that ethics review was not necessary for this study? Please include a copy of the correspondence as an ""Other"" file.

3. We note that you have indicated that there are restrictions to data sharing for this study. PLOS only allows data to be available upon request if there are legal or ethical restrictions on sharing data publicly. For more information on unacceptable data access restrictions, please see http://journals.plos.org/plosone/s/data-availability#loc-unacceptable-data-access-restrictions.

Before we proceed with your manuscript, please address the following prompts:

a) If there are ethical or legal restrictions on sharing a de-identified data set, please explain them in detail (e.g., data contain potentially identifying or sensitive patient information, data are owned by a third-party organization, etc.) and who has imposed them (e.g., a Research Ethics Committee or Institutional Review Board, etc.). Please also provide contact information for a data access committee, ethics committee, or other institutional body to which data requests may be sent.

b) If there are no restrictions, please upload the minimal anonymized data set necessary to replicate your study findings to a stable, public repository and provide us with the relevant URLs, DOIs, or accession numbers. For a list of recommended repositories, please see

https://journals.plos.org/plosone/s/recommended-repositories. You also have the option of uploading the data as Supporting Information files, but we would recommend depositing data directly to a data repository if possible.

We will update your Data Availability statement on your behalf to reflect the information you provide.

4. Please upload a new copy of Figures 1 to 9 as the detail is not clear. Please follow the link for more information:  https://journals.plos.org/plosone/s/figures

5. If the reviewer comments include a recommendation to cite specific previously published works, please review and evaluate these publications to determine whether they are relevant and should be cited. There is no requirement to cite these works unless the editor has indicated otherwise.

**Additional Editor Comments:**

Major revision is needed.

[Note: HTML markup is below. Please do not edit.]

Reviewers' comments:

Reviewer's Responses to Questions

**Comments to the Author**

1. Is the manuscript technically sound, and do the data support the conclusions?

Reviewer #1: Partly

Reviewer #2: Partly

Reviewer #3: Yes

**********

2. Has the statistical analysis been performed appropriately and rigorously?

Reviewer #1: No

Reviewer #2: Yes

Reviewer #3: Yes

**********

3. Have the authors made all data underlying the findings in their manuscript fully available?

The PLOS Data policy

Reviewer #1: No

Reviewer #2: No

Reviewer #3: Yes

**********

4. Is the manuscript presented in an intelligible fashion and written in standard English?

Reviewer #1: Yes

Reviewer #2: Yes

Reviewer #3: Yes

**********

Reviewer #1: The authors studied five non-invasive cardiac signals (ECG, PCG, SCG, PPG, PiPG) in 20 healthy subjects and provides a comparative time and frequency domain characterization. I have some comments:

1) The paper largely confirms known physiological delays and frequency bands without offering new physiological insights, algorithmic advances, or clinically actionable thresholds. A simple descriptive study in 20 young healthy adults under resting conditions does not constitute a generalizable reference dataset.

2) N=20 is small for establishing population wide benchmarks. No power calculation is provided. The study includes only healthy subjects, severely limiting generalizability to real world ambulatory monitoring.

3) Aligning each cardiac cycle to the maximum amplitude within a predefined window artificially sharpens waveforms and reduces true temporal variability. This biases correlation and delay estimates. The median waveform computed after per cycle peak alignment is not a true ensemble average and cannot be directly compared with standard ECG averaging methods.

4) DC subtraction after windowing is appropriate, but the paper does not describe handling of baseline wander, motion artifacts, or outlier beat rejection.

5) At HR > 75 bpm, diastole shortens, yet the same PCG S2 window [+280, +500] is used for all subjects – a systematic error. PPG window [+150, +1350] corresponds to HR ≥ 45 bpm, but the study did not report actual HR ranges. No justification is given for choosing a 600 ms refractory period for R peak detection to favor specificity (this may discard real beats in subjects with resting HR near 100 bpm).

6) Power spectral density (PSD) is estimated on individual pulse windows (not long continuous segments). This is unusual and discards low frequency HRV information (<0.5 Hz).

7) Intra vs. inter subject comparisons are presented without formal statistical testing.

8) Pearson’s r is used to compare waveform morphology after amplitude normalization and temporal alignment to the peak. This inflates correlation.

Reviewer #2: This manuscript presents a time and frequency domain comparison of five noninvasive cardiac sensing modalities, namely ECG, PCG, SCG, PPG, and PiPG, under a synchronized acquisition and unified reference framework. The topic is of practical relevance. A key strength of the study lies in its synchronized multimodal acquisition design, and the manuscript provides a summary of temporal delays, waveform similarity, and spectral characteristics, which gives it potential reference value for multimodal cardiac signal processing and wearable monitoring system design. However, in its current form, the manuscript remains largely descriptive, and the methodological rigor and strength of evidence are still insufficient. In addition, some conclusions appear to extend beyond what is directly supported by the presented data. Overall, the study has publication potential, but the current version still requires further improvement in methodological clarification, interpretation of results, and overall consistency of presentation. For these reasons, I recommend major revision.

Main comments

1.The study is useful, but its novelty is mainly integrative rather than strongly methodological.

The main contribution is the unified comparison of several heartbeat sensing modalities under one system, rather than a new sensing principle, algorithm, or physiological model. That positioning is acceptable, but the manuscript should state it more clearly and avoid overstating its general significance.

2. The alignment strategy may improve visual consistency, but it may also bias waveform similarity and delay interpretation.

The manuscript states that signal-specific waves were extracted around local maxima within predefined windows, and that delays were artificially nullified for visualization. The authors also acknowledge that this may artificially sharpen the resulting waveform, especially for more variable signals such as PCG and SCG. This issue should be more carefully discussed, because some of the reported consistency may partly come from the processing strategy itself.

3. The conclusions should be more cautious because the results are system-dependent and based on a limited experimental setting.

The frequency-domain findings are influenced by hardware response, and this is particularly relevant because the PCG sensor description and the reported dominant PCG frequency range raise questions about how much of the spectral result reflects physiology versus instrumentation. In addition, the study only includes 20 healthy adults under resting conditions, and the statistical analysis is mainly descriptive. Therefore, the conclusions should be framed more explicitly as baseline observations under controlled conditions, rather than broadly generalizable modality-level claims.

Reviewer #3: This manuscript systematically compares the time’s frequency characteristics of five noninvasive cardiac signals aligned to the ECG R-peak. While the unified framework provides practical guidelines for multimodal monitoring, revisions are needed to address limited sample diversity, the rationale for static windowing, and hardware induced spectral constraints.

Q1: The study only includes 20 healthy adult volunteers aged 24 to 52. To make the proposed signal processing guidelines truly robust, the authors should discuss how these time’s frequency characteristics might shift in older populations or patients with cardiovascular pathologies.

Q2: A fixed refractory period of 600 ms was used for ECG R-peak detection. While appropriate for resting conditions, the authors should address how this algorithm could be dynamically adapted for ambulatory or exercise monitoring where HR frequently exceeds 100 bpm.

Q3: The extraction windows for PCG and SCG are defined using fixed ranges. Given that systolic and diastolic durations vary non linearly with heart rate, relying on static time windows relative to the R-peak might cause truncation or overlap at higher heart rates. This limitation should be explicitly discussed.

Q4: The ECG is bandpass filtered at 5-15 Hz before peak detection. However, the paper later states the peak frequency for QRS is 11 Hz with a bandwidth up to 18 Hz. The authors should confirm that the 15 Hz low-pass cutoff did not artificially attenuate the QRS complex or induce phase shifts affecting the exact latency measurements.

Q5: Table 1 shows substantial inter-subject variability for SCG. The authors attribute this to "sensor coupling and anatomical factors", but a more detailed analysis on whether body mass index or gender influenced this mechanical signal attenuation would add significant clinical value.

Q6: The spectral peaks for PCG were both found to be around 30~31 Hz. Physiological literature often notes differences in frequency content between the mitral or tricuspid closure and pulmonary closure . The authors should comment on why their analysis did not capture this expected distinction.

Q7: A sampling rate of 1000 Hz is used for all signals. While sufficient for ECG and PPG, PCG components can extend up to several hundred Hertz. The authors should clearly state that 1000 Hz might limit the analysis of higher acoustic harmonics.

Q8: The PPG wave peaks around 378 ms post R-peak. The authors should mention whether their evaluation considered the subjects' heights or arm lengths, as these anatomical dimensions directly dictate the physical Pulse Transit Time delay to the fingertip.

Q9: Data was collected under resting conditions to minimize artifacts. A discussion on how typical motion artifacts specifically affect the distinct frequency bands of PiPG vs. PPG is recommended.

**********

what does this mean?). If published, this will include your full peer review and any attached files.

If you choose “no”, your identity will remain anonymous but your review may still be made public.

**Do you want your identity to be public for this peer review?**  For information about this choice, including consent withdrawal, please see our Privacy Policy

Reviewer #1: No

Reviewer #2: No

Reviewer #3: No

**********

[NOTE: If reviewer comments were submitted as an attachment file, they will be attached to this email and accessible via the submission site. Please log into your account, locate the manuscript record, and check for the action link "View Attachments". If this link does not appear, there are no attachment files.]

To ensure your figures meet our technical requirements, please review our figure guidelines: https://journals.plos.org/plosone/s/figures

You may also use PLOS’s free figure tool, NAAS, to help you prepare publication quality figures: https://journals.plos.org/plosone/s/figures#loc-tools-for-figure-preparation.

NAAS will assess whether your figures meet our technical requirements by comparing each figure against our figure specifications.

# Author comment: Author response to Decision Letter 1

**DOI:** 10.1371/journal.pone.0357922.r002

16 Jun 2026

Dear Dr. Ming Zhang and Reviewers,

We would like to thank the editorial board and the reviewers for their constructive comments and suggestions regarding our manuscript. We have carefully addressed each point raised and have revised the manuscript accordingly.

Below is a point-by-point response to the reviewers' comments.

Please note that all line numbers mentioned in our responses refer to the "Revised Manuscript with Track Changes" version of the document

Response to Journal Requirements

1) Please ensure that your manuscript meets PLOS ONE's style requirements, including those for file naming.

We have updated the manuscript to strictly adhere to PLOS ONE’s formatting guidelines, including section headings, font requirements, and file naming conventions. All figures have been tested with the NAAS tool.

2) You indicated that ethical approval was not necessary for your study. We understand that the framework for ethical oversight requirements for studies of this type may differ depending on the setting and we would appreciate some further clarification regarding your research. Could you please provide further details on why your study is exempt from the need for approval and confirmation from your institutional review board or research ethics committee (e.g., in the form of a letter or email correspondence) that ethics review was not necessary for this study? Please include a copy of the correspondence as an ""Other"" file.

The study is a technical signal processing benchmark and does not seek to extend biological or medical knowledge. Under French regulation (Loi Jardé), research that focuses on technical performance rather than clinical outcomes is not considered a clinical study involving the human person (RIPH) and is therefore exempt from formal Institutional Review Board (IRB) approval (Deplanque et al., 2017). This legal framework distinguishes between biomedical research and technical evaluations that do not impact patient care or medical theory.

To support this, we have attached an email correspondence from Mathilde Herbet, our referent Clinical Research Associate at the Clinical Investigation Center (CIC-IT). For your convenience, here is the English translation of the provided email:

"I confirm that if the data collected for this research does not allow the person to be identified, directly or indirectly (no date, time, location, existing correspondence list), the GDPR does not apply because the data is completely anonymous. No declaration is necessary."

3)We note that you have indicated that there are restrictions to data sharing for this study. PLOS only allows data to be available upon request if there are legal or ethical restrictions on sharing data publicly. For more information on unacceptable data access restrictions, please see http://journals.plos.org/plosone/s/data-availability#loc-unacceptable-data-access-restrictions.

Before we proceed with your manuscript, please address the following prompts:

a) If there are ethical or legal restrictions on sharing a de-identified data set, please explain them in detail (e.g., data contain potentially identifying or sensitive patient information, data are owned by a third-party organization, etc.) and who has imposed them (e.g., a Research Ethics Committee or Institutional Review Board, etc.). Please also provide contact information for a data access committee, ethics committee, or other institutional body to which data requests may be sent.

b) If there are no restrictions, please upload the minimal anonymized data set necessary to replicate your study findings to a stable, public repository and provide us with the relevant URLs, DOIs, or accession numbers. For a list of recommended repositories, please see

https://journals.plos.org/plosone/s/recommended-repositories. You also have the option of uploading the data as Supporting Information files, but we would recommend depositing data directly to a data repository if possible.

We will update your Data Availability statement on your behalf to reflect the information you provide.

In accordance with PLOS ONE’s data availability policy, we have provided the minimal anonymized data set necessary to replicate the study findings. This data set, including individual temporal and spectral characteristics, is uploaded as a Supporting_Information_file.xlsx. There are no further ethical or legal restrictions preventing the sharing of these de-identified data.

4) Please upload a new copy of Figures 1 to 9 as the detail is not clear. Please follow the link for more information: https://journals.plos.org/plosone/s/figures

We have updated the resolution of all figures to comply with the dpi requirement. All files have been successfully tested and validated using the PLOS NAAS tool. The resulting high-resolution TIFF files have been uploaded to the submission system.

Response to Reviewer #1

1) The paper largely confirms known physiological delays and frequency bands without offering new physiological insights, algorithmic advances, or clinically actionable thresholds. A simple descriptive study in 20 young healthy adults under resting conditions does not constitute a generalizable reference dataset.

We acknowledge that N=20 is a modest sample size for a clinical trial. However, we would like to clarify that this work is primarily a descriptive technical analysis aimed at optimizing signal processing frameworks rather than establishing universal clinical norms. Since the study was conducted on a homogeneous group of healthy volunteers, the inter-subject physiological variability remained low.

2) N=20 is small for establishing population wide benchmarks. No power calculation is provided. The study includes only healthy subjects, severely limiting generalizability to real world ambulatory monitoring.

We contend that the statistical unit of interest in this context is the cardiac waveform itself. Our analysis is based on several validated cardiac cycles per modality, providing high confidence in the stability of the reported morphological and spectral templates. We have updated the Discussion (Lines 334 – 342) to reflect this perspective and to acknowledge that while N=20 is sufficient for this technical benchmark, larger cohorts are required for broader clinical generalizability.

3) Aligning each cardiac cycle to the maximum amplitude within a predefined window artificially sharpens waveforms and reduces true temporal variability. This biases correlation and delay estimates. The median waveform computed after per cycle peak alignment is not a true ensemble average and cannot be directly compared with standard ECG averaging methods.

We acknowledge the reviewer's point that aligning waveforms on local maxima (within a ±20 ms window) may "sharpen" the resulting templates and potentially inflate Pearson’s correlation coefficients. However, we contend that this strategy is methodologically necessary for multimodal analysis.

The delay between the electrical activation (ECG R-peak) and the subsequent mechanical events (PCG, SCG, or PPG) is subject to physiological "jitter" or intra-individual variability, influenced by factors such as instantaneous heart rate, autonomic tone, and respiratory phase. Without this local alignment, the ensemble averaging process would act as a low-pass filter, blurring the rapid mechanical transients (like the S1 sound or SCG waves) and misrepresenting their true morphology.

By allowing for this fine-tuning, we compensate for these minor physiological fluctuations to capture the most representative "typical" waveform for each modality. We have added a comment in the Methods section (Lines 175 – 183) to clarify this trade-off between alignment precision and the resulting morphological consistency.

4) DC subtraction after windowing is appropriate, but the paper does not describe handling of baseline wander, motion artifacts, or outlier beat rejection.

We have refined the description of the signal preprocessing in the Methods section to provide more clarity. Baseline wander was handled using the 5 Hz high-pass component of our bandpass filter. Furthermore, we have detailed the outlier rejection used in our analysis, where beats with R-R intervals falling outside 2.5 standard deviations from the median were excluded (Lines 125 – 131).

5) At HR > 75 bpm, diastole shortens, yet the same PCG S2 window [+280, +500] is used for all subjects – a systematic error. PPG window [+150, +1350] corresponds to HR ≥ 45 bpm, but the study did not report actual HR ranges. No justification is given for choosing a 600 ms refractory period for R peak detection to favor specificity (this may discard real beats in subjects with resting HR near 100 bpm).

We agree that a static window would introduce systematic errors at high heart rates. However, the subjects' heart rates remained stable and low throughout the recordings, with a mean of 62.3 bpm (range: 57.1–71.4 bpm). We have updated the Results section to report these HR ranges (Lines 206– 207).

Regarding the 600 ms refractory period: as the maximum HR observed was 71.4 bpm (corresponding to an R-R interval of ~840 ms), this setting remained well below the actual physiological intervals, ensuring no real beats were discarded while maintaining high specificity against noise.

6) Power spectral density (PSD) is estimated on individual pulse windows (not long continuous segments). This is unusual and discards low frequency HRV information (<0.5 Hz).

We have clarified the PSD estimation strategy in the Methods section (Lines 175 – 183). The PSD was calculated on individual cardiac windows specifically to capture the spectral signature of the waveform morphology. By isolating the pulse from inter-beat intervals and continuous background noise, we ensure that the identified peak frequencies and bandwidths are directly related to the physiological "wave" of interest, providing better guidance for heartbeat detector optimization.

7) Intra vs. inter subject comparisons are presented without formal statistical testing.

We acknowledge the need for formal statistical validation to support our descriptive findings. We have now performed a Wilcoxon signed-rank test to compare intra-individual versus inter-individual Pearson correlation coefficients for each modality. We have updated Table 2 and Table 4 to include these p-values.

8) Pearson’s r is used to compare waveform morphology after amplitude normalization and temporal alignment to the peak. This inflates correlation.

We agree with the reviewer that synchronization and normalization mechanically increase the correlation coefficients. We have updated the Discussion section (Lines 334 - 342) to explicitly state that these preprocessing steps lead to an artificial inflation of Pearson’s compared to raw signals. We now clarify that while this approach is necessary to isolate and evaluate morphological consistency for detector design, it likely results in higher r values than would be observed without such alignment.

Response to Reviewer #2

1) The study is useful, but its novelty is mainly integrative rather than strongly methodological. The main contribution is the unified comparison of several heartbeat sensing modalities under one system, rather than a new sensing principle, algorithm, or physiological model. That positioning is acceptable, but the manuscript should state it more clearly and avoid overstating its general significance.

We have updated the Introduction (Lines 49 – 55) to more clearly define the study's scope and novelty. We now emphasize the integrative nature of our work, highlighting that the primary contribution is the unified comparison of five modalities recorded simultaneously under identical conditions. This clarifies that the study serves as a technical benchmark for multisensor fusion and standardized signal processing in healthy populations.

2) The alignment strategy may improve visual consistency, but it may also bias waveform similarity and delay interpretation. The manuscript states that signal-specific waves were extracted around local maxima within predefined windows, and that delays were artificially nullified for visualization. The authors also acknowledge that this may artificially sharpen the resulting waveform, especially for more variable signals such as PCG and SCG. This issue should be more carefully discussed, because some of the reported consistency may partly come from the processing strategy itself.

We agree with the reviewer that synchronization and normalization mechanically increase the correlation coefficients. We have updated the Discussion section (Lines 343 - 349) to explicitly state that these preprocessing steps lead to an artificial inflation of Pearson’s compared to raw signals. We now clarify that while this approach is necessary to isolate and evaluate morphological consistency for detector design, it likely results in higher r values than would be observed without such alignment.

3) The conclusions should be more cautious because the results are system-dependent and based on a limited experimental setting. The frequency-domain findings are influenced by hardware response, and this is particularly relevant because the PCG sensor description and the reported dominant PCG frequency range raise questions about how much of the spectral result reflects physiology versus instrumentation. In addition, the study only includes 20 healthy adults under resting conditions, and the statistical analysis is mainly descriptive. Therefore, the conclusions should be framed more explicitly as baseline observations under controlled conditions, rather than broadly generalizable modality-level claims.

We agree that the hardware response is a critical factor in spectral analysis. We have added the following technical information to the manuscript to address this:

• SCG: We added (Lines 75 – 76) the specific bandwidth of the ADXL356 (2400 Hz on the Z-axis) to the material description to show that the sensor did not limit the captured vibrations.

• PCG: We added (Lines 72-73) the bandwidth and noise performance of the TS472 preamplifier to the material description to confirm high-fidelity acoustic capture.

• Sampling Rate: We explicitly addressed (Lines 87 – 90) the 1000 Hz sampling rate limitation in the Methods and Discussion sections, clarifying that while it may limit high-frequency harmonic analysis, it is sufficient for the study's primary goal of heart rate detection optimization.

• Added Wilcoxon signed-rank tests (p-values) to Tables 2 and 4 to formally compare intra- vs. inter-subject correlations. This demonstrates that despite the small N=20, the observed morphological patterns are statistically significant.

We have clarified in the Discussion (Lines 301 – 307, 317 – 320 & 324 - 327) that these benchmarks are system-dependent and provide a reference for integrated monitoring systems using similar noninvasive technologies.

Response to Reviewer #3

1) The study only includes 20 healthy adult volunteers aged 24 to 52. To make the proposed signal processing guidelines truly robust, the authors should discuss how these time’s frequency characteristics might shift in older populations or patients with cardiovascular pathologies.

We have updated the discussion section (Lines 334 – 342) to discuss population diversity and physiological factors. We explicitly acknowledge that BMI, which was not recorded, likely influences the attenuation of mechanical vibrations (SCG/PCG) through adipose tissue. We also discussed how age and cardiovascular pathologies would shift the observed benchmarks due to changes in arterial stiffness and cardiac mechanics.

2) A fixed refractory period of 600 ms was used for ECG R-peak detection. While appropriate for resting conditions, the authors should address how this algorithm could be dynamically adapted for ambulatory or exercise monitoring where HR frequently exceeds 100 bpm.

We agree that a fixed 600 ms refractory period is a limitation for high heart rate monitoring. However, in the context of this resting-state study, the heart rates remained between 57.1 and 71.4 bpm (mean: 62.3 ± 4.2 bpm).

With a maximum observed heart rate of 71.4 bpm, the minimum R-R interval was approximately 840 ms, leaving a safety margin of 240 ms

Attachment Submitted filename: Response to Reviewers.docx

# Peer review: Decision Letter 1

**DOI:** 10.1371/journal.pone.0357922.r003

**Authors:** Ming Zhang

15 Jul 2026

Dear Dr. CHARLIER,

Please submit your revised manuscript by Aug 29 2026 11:59PM. If you will need more time than this to complete your revisions, please reply to this message or contact the journal office at plosone@plos.org. When you're ready to submit your revision, log on to https://www.editorialmanager.com/pone/ and select the 'Submissions Needing Revision' folder to locate your manuscript file.

- A letter that responds to each point raised by the academic editor and reviewer(s). You should upload this letter as a separate file labeled 'Response to Reviewers'.
- A marked-up copy of your manuscript that highlights changes made to the original version. You should upload this as a separate file labeled 'Revised Manuscript with Track Changes'.
- An unmarked version of your revised paper without tracked changes. You should upload this as a separate file labeled 'Manuscript'.

If you would like to make changes to your financial disclosure, please include your updated statement in your cover letter. Guidelines for resubmitting your figure files are available below the reviewer comments at the end of this letter.

If applicable, we recommend that you deposit your laboratory protocols in protocols.io to enhance the reproducibility of your results. Protocols.io assigns your protocol its own identifier (DOI) so that it can be cited independently in the future. For instructions see: https://journals.plos.org/plosone/s/submission-guidelines#loc-laboratory-protocols. Additionally, PLOS ONE offers an option for publishing peer-reviewed Lab Protocol articles, which describe protocols hosted on protocols.io. Read more information on sharing protocols at https://plos.org/protocols?utm_medium=editorial-email&utm_source=authorletters&utm_campaign=protocols.

As the corresponding author, your ORCID iD is verified in the submission system and will appear in the published article. PLOS supports the use of ORCID, and we encourage all coauthors to register for an ORCID iD and use it as well. Please encourage your coauthors to verify their ORCID iD within the submission system before final acceptance, as unverified ORCID iDs will not appear in the published article. *Only*  the individual author can complete the verification step; PLOS staff *cannot*  verify ORCID iDs on behalf of authors.

We look forward to receiving your revised manuscript.

Kind regards,

Ming Zhang

Academic Editor

PLOS One

Journal Requirements:

If the reviewer comments include a recommendation to cite specific previously published works, please review and evaluate these publications to determine whether they are relevant and should be cited. There is no requirement to cite these works unless the editor has indicated otherwise.

Please review your reference list to ensure that it is complete and correct. If you have cited papers that have been retracted, please include the rationale for doing so in the manuscript text, or remove these references and replace them with relevant current references. Any changes to the reference list should be mentioned in the rebuttal letter that accompanies your revised manuscript. If you need to cite a retracted article, indicate the article’s retracted status in the References list and also include a citation and full reference for the retraction notice.

Additional Editor Comments :

Dear authors,

Thank you for submitting your paper to PLOS One. We have received the reviews on your manuscript. The reviewers have identified several areas of concern; however, they also see potential in your work. Therefore, I am pleased to invite you to submit a minor revision of your manuscript.

Please carefully address all the comments and concerns raised by the reviewers in your revised version. You should also provide a point-by-point response letter explaining how you have addressed each comment.

We look forward to receiving your revised manuscript. Please note that the revised version will be sent back to the reviewers for further evaluation.

Best wishes.

Ming Zhang, PhD

Academic Editor at PLOS One

[Note: HTML markup is below. Please do not edit.]

Reviewers' comments:

Reviewer's Responses to Questions

**Comments to the Author**

Reviewer #1: All comments have been addressed

Reviewer #2: All comments have been addressed

Reviewer #3: (No Response)

**********

2. Is the manuscript technically sound, and do the data support the conclusions?

Reviewer #1: Yes

Reviewer #2: Yes

Reviewer #3: Partly

**********

3. Has the statistical analysis been performed appropriately and rigorously?

Reviewer #1: Yes

Reviewer #2: Yes

Reviewer #3: Yes

**********

4. Have the authors made all data underlying the findings in their manuscript fully available?

The PLOS Data policy

Reviewer #1: Yes

Reviewer #2: Yes

Reviewer #3: Yes

**********

5. Is the manuscript presented in an intelligible fashion and written in standard English?

Reviewer #1: Yes

Reviewer #2: Yes

Reviewer #3: Yes

**********

Reviewer #1: I approve revised paper. The authors have responded to my comments quite satisfactorily and have made the necessary corrections to the text of the article.

Reviewer #2: The authors have addressed my concerns as well as the concerns from other reviewers. I don't have any addtional comments.

Reviewer #3: The manuscript presents a simultaneous time- and frequency-domain comparison of ECG, PCG, SCG, PPG, and PiPG signals. The synchronized multimodal acquisition is potentially useful, but important ethical, signal-processing, statistical, and reproducibility issues must be addressed before the conclusions can be considered reliable.

1. The simultaneous acquisition of five cardiac signal modalities is a strength. However, the manuscript should clarify whether its main contribution is a physiological comparison, a hardware-validation study, a signal-processing benchmark, or a publicly reusable reference dataset.

2. The sample is limited to 20 healthy adults recorded for five minutes under resting conditions. No sample-size justification is provided. Claims regarding general modality characteristics, wearable monitoring, and standardization should therefore be restricted to this small, healthy, resting cohort.

3. The reported spectra may reflect the specific sensors and analog front-end rather than the physiological modalities themselves. Full frequency-response curves, gains, hardware filters, anti-aliasing procedures, calibration information, and noise floors should be reported for every acquisition channel.

4. The microphone is described as having a stable response from 50 Hz, yet the reported dominant PCG frequencies are approximately 30–31 Hz. The authors must demonstrate that these low-frequency measurements are reliable after considering the complete microphone–preamplifier–ADC transfer function.

5. The alignment procedure may artificially increase waveform similarity. Individual signals are centered on local maxima within predefined windows, and the manuscript acknowledges that this may sharpen median waveforms. Results should be compared with an ECG-only alignment analysis to quantify the bias introduced by modality-specific re-centering.

6. Inter-subject similarity appears to compare each participant with a grand median that includes that participant’s own waveform. This creates circularity and may inflate correlations. A leave-one-subject-out reference waveform should be used for each participant.

7. Pearson correlation alone is insufficient for assessing reproducibility because it is affected by alignment and is insensitive to amplitude differences after normalization. The authors should supplement it with confidence intervals and appropriate measures of timing, morphology, and agreement, such as latency variability, normalized error, dynamic time warping, or intraclass correlation.

8. Welch PSD is applied to relatively short event windows using a 2048-point FFT, which likely involves extensive zero-padding. The authors should report the actual segment duration, effective frequency resolution, detrending and windowing procedures, and sensitivity of peak-frequency estimates to analysis settings.

9. Since the study emphasizes multimodal sensing and signal integration, the authors may briefly discuss broader advances in multimodal integrated sensing systems. For reference, see DOI: 10.1109/MCOM.004.2400281. Such discussion should remain secondary to directly relevant biomedical sensor-fusion literature.

10. The physiological interpretation of the spectral peaks should be more cautious. For PPG and PiPG, the dominant 1–2 Hz component may largely reflect the participant’s heart rate rather than an intrinsic modality-specific bandwidth. Analyses separating the fundamental cardiac frequency from waveform harmonics would be more informative.

11. SCG1 and SCG2 are defined by their temporal correspondence with PCG windows rather than established SCG fiducial events. Without an independent mechanical or imaging reference, the manuscript should avoid implying that these peaks directly identify specific valve events.

12. The statistical analysis is mostly descriptive, yet terms such as significant similarity are used. The authors should report confidence intervals, participant-level distributions, paired comparisons across modalities, and sensitivity analyses. The numbers of excluded beats, failed detections, motion artifacts, and manually corrected peaks should also be disclosed.

**********

what does this mean?). If published, this will include your full peer review and any attached files.

If you choose “no”, your identity will remain anonymous but your review may still be made public.

**Do you want your identity to be public for this peer review?**  For information about this choice, including consent withdrawal, please see our Privacy Policy

Reviewer #1: No

Reviewer #2: No

Reviewer #3: No

**********

[NOTE: If reviewer comments were submitted as an attachment file, they will be attached to this email and accessible via the submission site. Please log into your account, locate the manuscript record, and check for the action link "View Attachments". If this link does not appear, there are no attachment files.]

To ensure your figures meet our technical requirements, please review our figure guidelines: https://journals.plos.org/plosone/s/figures

You may also use PLOS’s free figure tool, NAAS, to help you prepare publication quality figures: https://journals.plos.org/plosone/s/figures#loc-tools-for-figure-preparation.

NAAS will assess whether your figures meet our technical requirements by comparing each figure against our figure specifications.

# Author comment: Author response to Decision Letter 2

**DOI:** 10.1371/journal.pone.0357922.r004

20 Jul 2026

Dear Dr. Ming Zhang and Reviewers,

Thank you for the opportunity to submit a revised version of our manuscript, "Time and Frequency Characteristics of various noninvasive heart beat Sensors". We appreciate the time and effort the reviewers dedicated to providing insightful feedback. We have addressed all concerns, which have significantly strengthened our paper.

Below is our point-by-point response to the reviewers' comments.

Reviewer #1 and Reviewer #2

We thank both reviewers for their positive evaluation and for approving the manuscript.

Reviewer #3

1) The simultaneous acquisition of five cardiac signal modalities is a strength. However, the manuscript should clarify whether its main contribution is a physiological comparison, a hardware-validation study, a signal-processing benchmark, or a publicly reusable reference dataset.

We agree with the reviewer and have revised the last paragraph of the Introduction to clarify our main contribution. We explicitly state that this work aims to provide a "signal-processing benchmark and physiological comparison" rather than a "hardware-validation study".

2) The sample is limited to 20 healthy adults recorded for five minutes under resting conditions. No sample-size justification is provided. Claims regarding general modality characteristics, wearable monitoring, and standardization should therefore be restricted to this small, healthy, resting cohort.

We agree with this observation. We have updated the Conclusion section to explicitly restrict our claims regarding general modality characteristics and standardization to the specific cohort studied (a small cohort of healthy adults under resting conditions).

3) The reported spectra may reflect the specific sensors and analog front-end rather than the physiological modalities themselves. Full frequency-response curves, gains, hardware filters, anti-aliasing procedures, calibration information, and noise floors should be reported for every acquisition channel.

We have updated the Methods section to explicitly report these hardware parameters based on the analog front-end specifications. We added details specifying its high linearity, the input-referred noise floor and clarified that anti-aliasing is managed by the device.

4) The microphone is described as having a stable response from 50 Hz, yet the reported dominant PCG frequencies are approximately 30–31 Hz. The authors must demonstrate that these low-frequency measurements are reliable after considering the complete microphone–preamplifier–ADC transfer function.

We thank the reviewer for this important comment. We acknowledge that the manufacturer specifies the microphone operating frequency range from 50 Hz to 16 kHz and does not provide a calibrated frequency response below 50 Hz. We therefore cannot claim a fully characterized response in this frequency range. However, we performed internal measurements on the complete acquisition chain (microphone, preamplifier, and ADC), which demonstrated that the system remains sensitive to frequencies down to approximately 15 Hz, although the response is no longer perfectly linear. Consequently, the reported spectral components around 30 Hz should be interpreted as indicative of the dominant frequency content rather than as absolute calibrated amplitude measurements. We have clarified this limitation in the revised manuscript.

5) The alignment procedure may artificially increase waveform similarity. Individual signals are centered on local maxima within predefined windows, and the manuscript acknowledges that this may sharpen median waveforms. Results should be compared with an ECG-only alignment analysis to quantify the bias introduced by modality-specific re-centering.

We thank the reviewer for this remark. While an ECG-only alignment is a standard approach, it is physiologically suboptimal for this study. As shown by our results in Table 3, we observed significant intra-individual timing jitter (standard deviations ranging from ±17.53 ms for PiPG to ±48.24 ms for PPG). Relying on a fixed ECG-only alignment over 5-minute recordings would result in a severe low-pass filtering effect, which would completely deform the median waveform morphology and obscure rapid mechanical transients. We maintain that the local maximum recentering is a necessary procedure to compensate for this natural physiological variability and to preserve the true morphology of the signals; justification is present in the materials & methods section.

6) Inter-subject similarity appears to compare each participant with a grand median that includes that participant’s own waveform. This creates circularity and may inflate correlations. A leave-one-subject-out reference waveform should be used for each participant.

We thank the reviewer for this observation. We agree that the use of a grand median including the subject under evaluation may introduce a small degree of circularity and potentially overestimate inter-subject similarity. To address this concern, the inter-subject analysis was revised using a leave-one-subject-out approach. For each participant, the reference waveform was recomputed from all remaining participants, excluding the waveform of the subject being evaluated. The corresponding correlation values were recalculated and the manuscript has been updated accordingly.

7) Pearson correlation alone is insufficient for assessing reproducibility because it is affected by alignment and is insensitive to amplitude differences after normalization. The authors should supplement it with confidence intervals and appropriate measures of timing, morphology, and agreement, such as latency variability, normalized error, dynamic time warping, or intraclass correlation.

We thank the reviewer for this valuable comment. We agree that additional agreement metrics such as latency variability, normalized error, dynamic time warping, or intraclass correlation can provide complementary information depending on the study objective. In the present work, however, our primary objective was to provide a descriptive characterization of the reproducibility of the waveform morphology for signal processing applications rather than to perform a comprehensive method-comparison study. Since all signals were preprocessed and amplitude-normalized prior to analysis, absolute amplitude differences were not considered relevant, as they are highly dependent on the sensing modality, sensor characteristics, and placement rather than on the underlying physiological event. For this reason, Pearson correlation was selected to quantify waveform reproducibility after normalization. We acknowledge that additional agreement metrics could further complement the analysis and will consider including them in future work.

8) Welch PSD is applied to relatively short event windows using a 2048-point FFT, which likely involves extensive zero-padding. The authors should report the actual segment duration, effective frequency resolution, detrending and windowing procedures, and sensitivity of peak-frequency estimates to analysis settings.

We thank the reviewer for this insightful comment. We have clarified the spectral analysis methodology in the revised manuscript by specifying the segment duration, frequency resolution, detrending procedure, windowing parameters, and FFT settings. Specifically, the Welch PSD analysis was performed on isolated cardiac peak windows with durations ranging from 200 ms to 400 ms, resulting in an intrinsic frequency resolution of 2.5 to 5 Hz. Each segment was detrended by subtracting its mean value to remove DC bias, and a Hamming window with 50% overlap was applied using the MATLAB pwelch function. A 2048-point FFT was used to provide finer spectral interpolation of the frequency domain without affecting the intrinsic frequency resolution. These details have been added to the revised Methods section to improve the reproducibility and clarity of the spectral analysis.

9) Since the study emphasizes multimodal sensing and signal integration, the authors may briefly discuss broader advances in multimodal integrated sensing systems. For reference, see DOI: 10.1109/MCOM.004.2400281. Such discussion should remain secondary to directly relevant biomedical sensor-fusion literature.

This publication aims to provide a descriptive analysis of various cardiac signal acquisition technologies in order to guide designers and developers in their technological choices regarding signal acquisition and processing techniques. This work therefore emphasizes sensors technologies description, rather than multimodal sensing which, in our view, constitutes a separate research topic.

10) The physiological interpretation of the spectral peaks should be more cautious. For PPG and PiPG, the dominant 1–2 Hz component may largely reflect the participant’s heart rate rather than an intrinsic modality-specific bandwidth. Analyses separating the fundamental cardiac frequency from waveform harmonics would be more informative.

We acknowledge the reviewer's point regarding the interpretation of spectral peaks. We have revised our discussion to clarify that for PPG and PiPG, the 1–2 Hz dominant component is essentially governed by the fundamental heart rate. However, we emphasize that since these signals are hemodynamic pulse waves, their usable bandwidth is physiologically tied to the cardiac cycle duration. We have added a statement in the Discussion specifying that for these modalities, the spectral content is inherently linked to the patient's heart rate, which justifies this frequency range as the primary observation window.

11) SCG1 and SCG2 are defined by their temporal correspondence with PCG windows rather than established SCG fiducial events. Without an independent mechanical or imaging reference, the manuscript should avoid implying that these peaks directly identify specific valve events.

We completely agree. We have carefully reviewed the manuscript and removed any ambiguous phrasing that could imply these SCG peaks directly identify specific mechanical valve events. We ensured they are strictly defined by their temporal correspondence with the PCG windows.

We have reformulated the relevant section in the Methods to explicitly state that the terms SCG1 and SCG2 are used strictly based on their temporal correspondence with PCG windows, without implying that these components directly identify specific valve events.

12) The statistical analysis is mostly descriptive, yet terms such as significant similarity are used. The authors should report confidence intervals, participant-level distributions, paired comparisons across modalities, and sensitivity analyses. The numbers of excluded beats, failed detections, motion artifacts, and manually corrected peaks should also be disclosed.

We thank the reviewer for this comment. The objective of this study was primarily descriptive and focused on the characterization of the extracted physiological features and their similarity across sensing modalities under controlled resting conditions. Therefore, the statistical analysis was designed to provide descriptive metrics of waveform morphology and spectral consistency rather than to perform hypothesis-driven comparisons between modalities.

To improve transparency, we have added the number of excluded cardiac cycles resulting from the automated RR outlier rejection procedure. Across the 20 recordings, 6360 cardiac cycles were detected, and 171 cycles (2.7%) were excluded based on the predefined RR interval rejection criterion. No manual peak corrections were performed. Motion artifacts were not specifically quantified because the recordings were performed under controlled conditions, with all subjects resting during acquisition, resulting in minimal motion-related disturbances.

Sincerely,

Pierre Charlier, PhD

Corresponding Author

CHU Lille, France

Attachment Submitted filename: Response_to_Reviewers_auresp_2.docx

# Peer review: Decision Letter 2

**DOI:** 10.1371/journal.pone.0357922.r005

**Authors:** Ming Zhang

5 Aug 2026

Dear Dr. CHARLIER,

Please submit your revised manuscript by Sep 19 2026 11:59PM. If you will need more time than this to complete your revisions, please reply to this message or contact the journal office at plosone@plos.org. When you're ready to submit your revision, log on to https://www.editorialmanager.com/pone/ and select the 'Submissions Needing Revision' folder to locate your manuscript file.

- A letter that responds to each point raised by the academic editor and reviewer(s). You should upload this letter as a separate file labeled 'Response to Reviewers'.
- A marked-up copy of your manuscript that highlights changes made to the original version. You should upload this as a separate file labeled 'Revised Manuscript with Track Changes'.
- An unmarked version of your revised paper without tracked changes. You should upload this as a separate file labeled 'Manuscript'.

If you would like to make changes to your financial disclosure, please include your updated statement in your cover letter. Guidelines for resubmitting your figure files are available below the reviewer comments at the end of this letter.

If applicable, we recommend that you deposit your laboratory protocols in protocols.io to enhance the reproducibility of your results. Protocols.io assigns your protocol its own identifier (DOI) so that it can be cited independently in the future. For instructions see: https://journals.plos.org/plosone/s/submission-guidelines#loc-laboratory-protocols. Additionally, PLOS ONE offers an option for publishing peer-reviewed Lab Protocol articles, which describe protocols hosted on protocols.io. Read more information on sharing protocols at https://plos.org/protocols?utm_medium=editorial-email&utm_source=authorletters&utm_campaign=protocols.

As the corresponding author, your ORCID iD is verified in the submission system and will appear in the published article. PLOS supports the use of ORCID, and we encourage all coauthors to register for an ORCID iD and use it as well. Please encourage your coauthors to verify their ORCID iD within the submission system before final acceptance, as unverified ORCID iDs will not appear in the published article. *Only*  the individual author can complete the verification step; PLOS staff *cannot*  verify ORCID iDs on behalf of authors.

We look forward to receiving your revised manuscript.

Kind regards,

Ming Zhang

Academic Editor

PLOS One

Journal Requirements:

If the reviewer comments include a recommendation to cite specific previously published works, please review and evaluate these publications to determine whether they are relevant and should be cited. There is no requirement to cite these works unless the editor has indicated otherwise.

Please review your reference list to ensure that it is complete and correct. If you have cited papers that have been retracted, please include the rationale for doing so in the manuscript text, or remove these references and replace them with relevant current references. Any changes to the reference list should be mentioned in the rebuttal letter that accompanies your revised manuscript. If you need to cite a retracted article, indicate the article’s retracted status in the References list and also include a citation and full reference for the retraction notice.

Additional Editor Comments :

Dear authors,

Thank you for submitting your paper. We have received the reviews on your manuscript. The reviewers have identified several areas of concern; however, they also see potential in your work. Therefore, I am pleased to invite you to submit a minor revision of your manuscript.

Please carefully address all the comments and concerns raised by the reviewers in your revised version. You should also provide a point-by-point response letter explaining how you have addressed each comment.

We look forward to receiving your revised manuscript. Please note that the revised version will be sent back to the reviewers for further evaluation.

Best wishes.

Ming Zhang, PhD

Academic Editor

[Note: HTML markup is below. Please do not edit.]

Reviewers' comments:

Reviewer's Responses to Questions

**Comments to the Author**

Reviewer #3: (No Response)

**********

2. Is the manuscript technically sound, and do the data support the conclusions?

Reviewer #3: Yes

**********

3. Has the statistical analysis been performed appropriately and rigorously?

Reviewer #3: Yes

**********

4. Have the authors made all data underlying the findings in their manuscript fully available?

The PLOS Data policy

Reviewer #3: Yes

**********

5. Is the manuscript presented in an intelligible fashion and written in standard English?

Reviewer #3: Yes

**********

Reviewer #3: The revised manuscript improves its positioning, methodological description, and interpretation of several findings.

1. The PCG frequency concern is only partially addressed. The authors state that internal testing showed sensitivity down to approximately 15 Hz, but no calibration protocol, transfer-function curve, reference source, uncertainty, or raw measurement is provided. The reported 30 Hz peak should remain explicitly device-specific and qualitative.

2. The requested alignment-control analysis was not performed. Instead, the authors justify local peak recentering while also acknowledging that normalization and alignment mechanically inflate correlations. A direct ECG-locked versus locally aligned comparison is still needed to quantify this effect.

3. The leave-one-subject-out reference analysis appropriately resolves the previous circularity concern. This is an important improvement to the inter-subject comparison.

4. The reproducibility analysis remains incomplete. The authors retain Pearson correlation alone and provide mean ± standard deviation, but no confidence intervals or complementary morphology/agreement metrics. Describing high correlations as strong physiological reproducibility is therefore still too broad.

5. Since the manuscript continues to emphasize multimodal integration and future sensor-fusion applications, a brief discussion of related multimodal sensing developments remains relevant. For reference, see DOI: 10.1109/MCOM.004.2400281. This should remain secondary to biomedical literature.

**********

what does this mean?). If published, this will include your full peer review and any attached files.

If you choose “no”, your identity will remain anonymous but your review may still be made public.

**Do you want your identity to be public for this peer review?**  For information about this choice, including consent withdrawal, please see our Privacy Policy

Reviewer #3: No

**********

[NOTE: If reviewer comments were submitted as an attachment file, they will be attached to this email and accessible via the submission site. Please log into your account, locate the manuscript record, and check for the action link "View Attachments". If this link does not appear, there are no attachment files.]

To ensure your figures meet our technical requirements, please review our figure guidelines: https://journals.plos.org/plosone/s/figures

You may also use PLOS’s free figure tool, NAAS, to help you prepare publication quality figures: https://journals.plos.org/plosone/s/figures#loc-tools-for-figure-preparation.

NAAS will assess whether your figures meet our technical requirements by comparing each figure against our figure specifications.

# Author comment: Author response to Decision Letter 3

**DOI:** 10.1371/journal.pone.0357922.r006

7 Aug 2026

Pierre Charlier

CHU Lille, CIC 1403

Lille, France

pierre1.charlier@chu-lille.fr

August 17, 2026

Subject: Submission PONE-D-26-10913

Dear Dr. Ming Zhang and Reviewers,

Below is our point-by-point response to the reviewers' comments.

Reviewer #3

1) The PCG frequency concern is only partially addressed. The authors state that internal testing showed sensitivity down to approximately 15 Hz, but no calibration protocol, transfer-function curve, reference source, uncertainty, or raw measurement is provided. The reported 30 Hz peak should remain explicitly device-specific and qualitative.

We fully agree with the reviewer's assessment. We have updated the Discussion section (methodological limitations) to explicitly state that because no formal calibration protocol or transfer-function curve was established below 50 Hz for this specific setup, the reported 30 Hz peak for the PCG signal must be considered explicitly device-specific and qualitative.

2) The requested alignment-control analysis was not performed. Instead, the authors justify local peak recentering while also acknowledging that normalization and alignment mechanically inflate correlations. A direct ECG-locked versus locally aligned comparison is still needed to quantify this effect.

We apologize for not including this quantitative comparison in the previous revision. We have now performed a strict ECG-locked alignment analysis without local peak recentering. As expected, without compensating for physiological timing jitter, the strict ECG-locked ensemble averaging mechanically reduced the correlation coefficients by an average of delta r = -0.47 for PCG and delta r = -0.50 for SCG (detailed comparison provided in Supporting Information). These quantified results have also been integrated into the Results and Methods sections of the revised manuscript. This confirms that while local re-centering inflates raw waveform similarity, it remains a necessary step to compensate for the flattening effect of physiological timing jitter and preserve the true morphology of the signals.

3) The leave-one-subject-out reference analysis appropriately resolves the previous circularity concern. This is an important improvement to the inter-subject comparison.

We thank the reviewer for this positive feedback and for validating this methodological improvement.

4) The reproducibility analysis remains incomplete. The authors retain Pearson correlation alone and provide mean ± standard deviation, but no confidence intervals or complementary morphology/agreement metrics. Describing high correlations as strong physiological reproducibility is therefore still too broad.

We acknowledge this limitation. We have updated Tables 2 and 4 to include the 95% Confidence Intervals (CI) for all Pearson correlation coefficients, calculated using the Fisher z-transformation. Finally, we have toned down our claims in the Discussion, replacing broad statements like "strong physiological reproducibility" with more measured terms such as "high morphological agreement."

5) Since the manuscript continues to emphasize multimodal integration and future sensor-fusion applications, a brief discussion of related multimodal sensing developments remains relevant. For reference, see DOI: 10.1109/MCOM.004.2400281. This should remain secondary to biomedical literature.

We have added a brief statement to the end of the Introduction acknowledging broader developments in multimodal integrated sensing systems, and we have incorporated the suggested reference (DOI: 10.1109/MCOM.004.2400281), while ensuring it remains secondary to the core biomedical literature.

Sincerely,

Pierre Charlier, PhD

Corresponding Author

Attachment Submitted filename: Response_to_Reviewers_auresp_3.docx

# Peer review: Decision Letter 3

**DOI:** 10.1371/journal.pone.0357922.r007

**Authors:** Ming Zhang

20 Aug 2026

Dear Dr. CHARLIER,

Thank you for submitting your manuscript to PLOS One. After careful consideration, we feel that it has merit but does not fully meet PLOS One’s publication criteria as it currently stands. Therefore, we invite you to submit a revised version of the manuscript that addresses the points raised during the review process.

Please submit your revised manuscript by Oct 04 2026 11:59PM. If you will need more time than this to complete your revisions, please reply to this message or contact the journal office at plosone@plos.org. When you're ready to submit your revision, log on to https://www.editorialmanager.com/pone/ and select the 'Submissions Needing Revision' folder to locate your manuscript file.

- A letter that responds to each point raised by the academic editor and reviewer(s). You should upload this letter as a separate file labeled 'Response to Reviewers'.
- A marked-up copy of your manuscript that highlights changes made to the original version. You should upload this as a separate file labeled 'Revised Manuscript with Track Changes'.
- An unmarked version of your revised paper without tracked changes. You should upload this as a separate file labeled 'Manuscript'.

If you would like to make changes to your financial disclosure, please include your updated statement in your cover letter. Guidelines for resubmitting your figure files are available below the reviewer comments at the end of this letter.

If applicable, we recommend that you deposit your laboratory protocols in protocols.io to enhance the reproducibility of your results. Protocols.io assigns your protocol its own identifier (DOI) so that it can be cited independently in the future. For instructions see: https://journals.plos.org/plosone/s/submission-guidelines#loc-laboratory-protocols. Additionally, PLOS One offers an option for publishing peer-reviewed Lab Protocol articles, which describe protocols hosted on protocols.io. Read more information on sharing protocols at https://plos.org/protocols?utm_medium=editorial-email&utm_source=authorletters&utm_campaign=protocols.

As the corresponding author, your ORCID iD is verified in the submission system and will appear in the published article. PLOS supports the use of ORCID, and we encourage all coauthors to register for an ORCID iD and use it as well. Please encourage your coauthors to verify their ORCID iD within the submission system before final acceptance, as unverified ORCID iDs will not appear in the published article. *Only*  the individual author can complete the verification step; PLOS staff *cannot*  verify ORCID iDs on behalf of authors.

We look forward to receiving your revised manuscript.

Kind regards,

Ming Zhang

Academic Editor

PLOS One

Journal Requirements:

If the reviewer comments include a recommendation to cite specific previously published works, please review and evaluate these publications to determine whether they are relevant and should be cited. There is no requirement to cite these works unless the editor has indicated otherwise.

Please review your reference list to ensure that it is complete and correct. If you have cited papers that have been retracted, please include the rationale for doing so in the manuscript text, or remove these references and replace them with relevant current references. Any changes to the reference list should be mentioned in the rebuttal letter that accompanies your revised manuscript. If you need to cite a retracted article, indicate the article’s retracted status in the References list and also include a citation and full reference for the retraction notice.

Additional Editor Comments:

Dear authors,

Thank you for submitting your paper. We have received the reviews on your manuscript. The reviewers have identified several areas of concern; however, they also see potential in your work. Therefore, I am pleased to invite you to submit a minor revision of your manuscript.

Please carefully address all the comments and concerns raised by the reviewers in your revised version. You should also provide a point-by-point response letter explaining how you have addressed each comment.

We look forward to receiving your revised manuscript. Please note that the revised version will be sent back to the reviewers for further evaluation.

Best wishes.

Academic Editor

[Note: HTML markup is below. Please do not edit.]

Reviewers' comments:

Reviewer's Responses to Questions

**Comments to the Author**

Reviewer #3: (No Response)

**********

2. Is the manuscript technically sound, and do the data support the conclusions?

Reviewer #3: Yes

**********

3. Has the statistical analysis been performed appropriately and rigorously?

Reviewer #3: Yes

**********

4. Have the authors made all data underlying the findings in their manuscript fully available?

The PLOS Data policy

Reviewer #3: Yes

**********

5. Is the manuscript presented in an intelligible fashion and written in standard English?

Reviewer #3: No

**********

Reviewer #3: The manuscript has been substantially improved in the current revision, and I appreciate the authors careful efforts in addressing the previous comments. The remaining concerns are relatively minor and mainly relate to presentation and formatting. I recommend that the authors conduct a final thorough check of the manuscript.

1. The manuscript would benefit from a careful overall formatting revision. Please check the consistency of capitalization in the title and section headings, as well as font style, line spacing, paragraph indentation, and spacing before and after headings throughout the manuscript.

2. The formatting of figures and tables should be further standardized. Please ensure that all figure/table captions follow a consistent style, that abbreviations and units are presented uniformly, and that the numbering and in-text citations of figures and tables are carefully checked.

3. The reference and citation formatting should be carefully revised. Please ensure that all references follow a consistent journal style, including author names, journal titles, volume/issue information, page ranges, DOI presentation, and in-text citation format. The authors are also encouraged to expand the reference list to approximately 30 relevant references, particularly by including more recent studies related to noninvasive cardiac sensing, wearable monitoring, multimodal physiological signals, and sensor fusion.

4. Recent studies in DOI: (10.1016/j.sigpro.2026.110584), (10.1016/j.measurement.2025.118915), (10.1109/SMC58881.2025.11342493), (10.1016/j.bspc.2026.110367), and (10.1016/j.neucom.2025.130151) provide relevant background for this work. Including these studies would strengthen the positioning and novelty discussion of this manuscript.

5. The overall presentation of the manuscript still requires careful formatting and proofreading. There are many inconsistencies in capitalization, hyphenation, spacing, and terminology throughout the manuscript. For example, the title uses inconsistent capitalization (“various”, “Sensors”), and “heart beat” would normally be written as “heartbeat”. Terms such as “R peak/R-peak”, “time domain/time-domain”, “inter-subject/intersubject”, and “intra-individual/intra individual” should also be standardized throughout the manuscript.

6. The formatting of physical quantities and units should be thoroughly standardized according to SI conventions. A space should be inserted between numerical values and units, e.g., “600 ms” instead of “600ms”, “20 ms” instead of “20ms”, and “11 Hz” instead of “11Hz”. Similar inconsistencies occur repeatedly in the Methods, Results, and Discussion. The notation “100 min-1” should preferably be typeset as “100 min⁻¹” or expressed consistently as bpm.

7. Please carefully verify the units associated with sensor specifications. For example, the SCG accelerometer noise density is reported as “80 μg/Hz”. Since noise density is commonly expressed with a square-root bandwidth term, the authors should verify whether the intended unit is μg/√Hz and correct it if necessary.

8. The figure quality and visual consistency need substantial improvement. Several figures contain very small labels, thin lines, low-contrast elements, and excessive blank space. This is particularly evident in Figs. 5–9. For example, the axes and labels in Fig. 5 are difficult to read, while Figs. 8 and 9 contain multiple stacked panels with relatively small fonts. All figures should use consistent font sizes, line widths, axis styles, and labeling conventions and should remain readable at the final journal column width.

9. The authors should avoid relying primarily on color to distinguish important graphical information. In Figs. 8 and 9, individual curves and median curves are mainly differentiated using grey and red. Please consider combining color with different line styles/line widths so that the figures remain interpretable in grayscale printing and for readers with color-vision deficiency. High-resolution or preferably vector-format graphics should also be provided.

10. The arrangement of figure files in the submitted reviewer PDF should be checked. Figure 9 appears before Figures 1–8 in the appended figure section. Even if the figures are uploaded separately during submission, the final reviewer file should ideally present them in numerical order to facilitate reading and cross-checking.

11. Figure captions should be standardized and made more self-contained. There are inconsistent styles such as “Fig 1:”, “Fig 7 :”, and similar spacing differences before colons. Captions should use one journal-compliant format consistently. In addition, captions for multi-panel or information-dense figures should explain the curves, colors, shaded regions, abbreviations, and axes sufficiently without requiring extensive consultation of the main text.

12. The tables would benefit from a more professional journal-style layout. Tables 1–5 currently use dense full gridlines and relatively compact formatting. The authors may consider a cleaner table design with fewer horizontal/vertical rules, consistent decimal alignment, units placed in column headings, and uniform footnote notation. In addition, terms such as “Broadband (-3 dB)” in Table 5 should be reconsidered; “−3 dB bandwidth” would be more technically conventional and consistent with the Methods section.

13. There are apparent inconsistencies between numerical values reported in the text and those presented in the tables, which should be carefully checked. For example, the Results text reports ECG, PPG, and PiPG correlations of approximately 0.91, 0.92, and 0.96, whereas Table 2 reports corresponding inter-individual values of 0.90, 0.90, and 0.96. Similar discrepancies appear in the spectral-domain results, where the text reports PCG S1/S2 values around 0.79/0.84 while Table 4 shows 0.78/0.83. These differences suggest that some parts of the text or tables may not have been fully synchronized after revision.

14. Statistical reporting should be made more precise and consistent. Tables 2 and 4 report essentially all comparisons simply as “p < 0.05”. If available, exact p-values would provide more informative statistical reporting. The authors should also clarify whether any correction for multiple comparisons was considered, given that several modalities are evaluated simultaneously. The formatting of confidence intervals should also be standardized, for example using en dashes consistently rather than mixed hyphen styles.

15. A careful language and typesetting check is still needed. Several obvious editorial issues remain, such as “reference.Then” without a space, “fingers Sensors positioning”, inconsistent capitalization of “Human Beings”, and phrases such as “presented Table 1”, “As illustrated Fig 6”, or “summarized Table 2”, where “in” is missing. These issues do not affect the scientific meaning but significantly reduce the professional appearance of the manuscript.

16. The reference section requires urgent verification. The manuscript cites references with numbers well beyond 8, including citations such as (12), (18), (19), (20), (23), (24), and (25), whereas the reference list visible in the submitted PDF appears to end at Reference 8 before the figure files begin. The authors should confirm that all cited references are included and that citation numbering is correct throughout the manuscript.

17. The reference formatting itself should also be standardized. Some entries contain “[Internet]”, access dates, “Available from:” URLs, and DOI information, whereas other references contain only DOI information. Please carefully check all references against the journal style and ensure consistent formatting of journal abbreviations, dates, page ranges, URLs, and DOIs.

18. The manuscript would benefit from a final consistency check of terminology used in figures, tables, and the main text. For example, “PCGS1/PCGS2”, “PCG (S1)/(S2)”, “SCG1/SCG2”, and “SCG (SCG1)/(SCG2)” are used in different locations. A single notation scheme should be adopted throughout all figures, tables, captions, and textual descriptions.

19. The manuscript should also ensure that all mandatory reporting statements are presented clearly and in journal-compliant form. In particular, the ethics/waiver statement should clearly identify the responsible authority or regulatory basis, and the availability of the underlying dataset should be explicitly stated, especially because the Abstract describes the outcome as a “reference dataset”.

**********

what does this mean?). If published, this will include your full peer review and any attached files.

If you choose “no”, your identity will remain anonymous but your review may still be made public.

**Do you want your identity to be public for this peer review?**  For information about this choice, including consent withdrawal, please see our Privacy Policy

Reviewer #3: No

**********

[NOTE: If reviewer comments were submitted as an attachment file, they will be attached to this email and accessible via the submission site. Please log into your account, locate the manuscript record, and check for the action link "View Attachments". If this link does not appear, there are no attachment files.]

To ensure your figures meet our technical requirements, please review our figure guidelines: https://journals.plos.org/plosone/s/figures

You may also use PLOS’s free figure tool, NAAS, to help you prepare publication quality figures: https://journals.plos.org/plosone/s/figures#loc-tools-for-figure-preparation.

NAAS will assess whether your figures meet our technical requirements by comparing each figure against our figure specifications.

# Author comment: Author response to Decision Letter 4

**DOI:** 10.1371/journal.pone.0357922.r008

21 Aug 2026

Pierre Charlier

CHU Lille, CIC 1403

Lille, France

pierre1.charlier@chu-lille.fr

August 21, 2026

Subject: Submission PONE-D-26-10913

Dear Dr. Ming Zhang and Reviewers,

Below is our point-by-point response to the reviewers' comments.

Reviewer #3

1) The manuscript would benefit from a careful overall formatting revision. Please check the consistency of capitalization in the title and section headings, as well as font style, line spacing, paragraph indentation, and spacing before and after headings throughout the manuscript.

We agree with the reviewer's assessment. We have standardized the font style and paragraph indentation throughout the text. Furthermore, we have verified the consistency of capitalization in the title and all section headings, and ensured uniform spacing before and after headings to comply with standard academic formatting.

2) The formatting of figures and tables should be further standardized. Please ensure that all figure/table captions follow a consistent style, that abbreviations and units are presented uniformly, and that the numbering and in-text citations of figures and tables are carefully checked.

We have carefully reviewed and standardized all figures and tables. Figure and table captions now follow a strictly uniform style (e.g., standardizing the "Fig X." prefix). We have also ensured that all abbreviations (such as wave components) and units (adhering to SI conventions) are presented uniformly across all visual elements and the main text. Finally, we verified the sequential numbering and corrected the in-text citations to ensure proper referencing throughout the manuscript.

3) The reference and citation formatting should be carefully revised. Please ensure that all references follow a consistent journal style, including author names, journal titles, volume/issue information, page ranges, DOI presentation, and in-text citation format. The authors are also encouraged to expand the reference list to approximately 30 relevant references, particularly by including more recent studies related to noninvasive cardiac sensing, wearable monitoring, multimodal physiological signals, and sensor fusion.

We have carefully revised the reference and citation formatting. To ensure strict compliance and consistency, we have completely rebuilt our bibliography using the Zotero reference management software, applying the Vancouver style as required by the journal. Furthermore, the bibliography has been updated and expanded to 30 references to include recent studies related to noninvasive cardiac sensing, wearable monitoring, and sensor fusion, as suggested by the reviewer.

4) Recent studies in DOI: (10.1016/j.sigpro.2026.110584), (10.1016/j.measurement.2025.118915), (10.1109/SMC58881.2025.11342493), (10.1016/j.bspc.2026.110367), and (10.1016/j.neucom.2025.130151) provide relevant background for this work. Including these studies would strengthen the positioning and novelty discussion of this manuscript.

We thank the reviewer for the suggested references regarding advanced signal processing and feature extraction. We have carefully evaluated them and integrated the most relevant regarding multi-feature fusion and robust signal processing architectures into our Introduction. Furthermore, we have expanded our bibliography with recent studies directly focused on multimodal cardiac monitoring, sensor fusion (ECG, SCG, PPG, and PiPG), and wearable physiological acquisition.

5) The overall presentation of the manuscript still requires careful formatting and proofreading. There are many inconsistencies in capitalization, hyphenation, spacing, and terminology throughout the manuscript. For example, the title uses inconsistent capitalization (“various”, “Sensors”), and “heart beat” would normally be written as “heartbeat”. Terms such as “R peak/R-peak”, “time domain/time-domain”, “inter-subject/intersubject”, and “intra-individual/intra individual” should also be standardized throughout the manuscript.

We apologize for these editorial oversights. We have thoroughly proofread the manuscript to resolve all formatting, typesetting, and terminology inconsistencies.

6) The formatting of physical quantities and units should be thoroughly standardized according to SI conventions. A space should be inserted between numerical values and units, e.g., “600 ms” instead of “600ms”, “20 ms” instead of “20ms”, and “11 Hz” instead of “11Hz”. Similar inconsistencies occur repeatedly in the Methods, Results, and Discussion. The notation “100 min-1” should preferably be typeset as “100 min⁻¹” or expressed consistently as bpm.

We have carefully reviewed the manuscript and standardized the formatting of physical quantities according to SI conventions. Spaces have now been systematically inserted between numerical values and their respective units (e.g., "600 ms", "11 Hz"). Furthermore, heart rate units are now consistently expressed as "bpm" throughout the text.

7) Please carefully verify the units associated with sensor specifications. For example, the SCG accelerometer noise density is reported as “80 μg/Hz”. Since noise density is commonly expressed with a square-root bandwidth term, the authors should verify whether the intended unit is μg/√Hz and correct it if necessary.

We thank the reviewer for spotting this typographical error. We have corrected the noise density unit to "80 μg/√Hz" in the Methods section. We also proactively corrected a similar unit error for the PCG preamplifier (10 nV/√Hz).

8) The figure quality and visual consistency need substantial improvement. Several figures contain very small labels, thin lines, low-contrast elements, and excessive blank space. This is particularly evident in Figs. 5–9. For example, the axes and labels in Fig. 5 are difficult to read, while Figs. 8 and 9 contain multiple stacked panels with relatively small fonts. All figures should use consistent font sizes, line widths, axis styles, and labeling conventions and should remain readable at the final journal column width.

We thank the reviewer for this valuable feedback. We have thoroughly revised and improved Figures 5 through 9 to enhance visual clarity and consistency. Specifically, we increased the font sizes for all axis labels and numbers, thickened the line widths, and adjusted the layouts to eliminate excessive blank space.

9) The authors should avoid relying primarily on color to distinguish important graphical information. In Figs. 8 and 9, individual curves and median curves are mainly differentiated using grey and red. Please consider combining color with different line styles/line widths so that the figures remain interpretable in grayscale printing and for readers with color-vision deficiency. High-resolution or preferably vector-format graphics should also be provided.

We have updated Figures 8 and 9 by combining color differentiation with distinct line widths and line styles. Furthermore, improved-resolution graphics have been provided for all figures

10) The arrangement of figure files in the submitted reviewer PDF should be checked. Figure 9 appears before Figures 1–8 in the appended figure section. Even if the figures are uploaded separately during submission, the final reviewer file should ideally present them in numerical order to facilitate reading and cross-checking.

We thank the reviewer for pointing this out. We will ensure that all figure files are uploaded in correct numerical order during the submission process.

11) Figure captions should be standardized and made more self-contained. There are inconsistent styles such as “Fig 1:”, “Fig 7 :”, and similar spacing differences before colons. Captions should use one journal-compliant format consistently. In addition, captions for multi-panel or information-dense figures should explain the curves, colors, shaded regions, abbreviations, and axes sufficiently without requiring extensive consultation of the main text.

We have standardized all figure captions to follow a consistent, journal-compliant format (e.g., "Fig 1."). Furthermore, we have expanded the captions for multi-panel and information-dense figures (specifically Figs 8 and 9) to fully explain the curves, colors, and reference axes, ensuring they are entirely self-contained without needing to consult the main text.

12) The tables would benefit from a more professional journal-style layout. Tables 1–5 currently use dense full gridlines and relatively compact formatting. The authors may consider a cleaner table design with fewer horizontal/vertical rules, consistent decimal alignment, units placed in column headings, and uniform footnote notation. In addition, terms such as “Broadband (-3 dB)” in Table 5 should be reconsidered; “−3 dB bandwidth” would be more technically conventional and consistent with the Methods section.

We have completely reformatted Tables 1–5 to adhere to a professional, journal-compliant layout. We removed all vertical rules, minimized horizontal rules, aligned decimals, and standardized footnote notations. Additionally, the term “Broadband (-3 dB)” has been updated to “−3 dB bandwidth” in Table 5 to ensure consistency with the Methods section.

13) There are apparent inconsistencies between numerical values reported in the text and those presented in the tables, which should be carefully checked. For example, the Results text reports ECG, PPG, and PiPG correlations of approximately 0.91, 0.92, and 0.96, whereas Table 2 reports corresponding inter-individual values of 0.90, 0.90, and 0.96. Similar discrepancies appear in the spectral-domain results, where the text reports PCG S1/S2 values around 0.79/0.84 while Table 4 shows 0.78/0.83. These differences suggest that some parts of the text or tables may not have been fully synchronized after revision.

We deeply apologize for these discrepancies, which resulted from an incomplete text update following our latest data reprocessing. We have carefully cross-checked all numerical values in the text against the tables. The manuscript text has been fully synchronized with the correct, definitive values presented in Tables 2 and 4.

14) Statistical reporting should be made more precise and consistent. Tables 2 and 4 report essentially all comparisons simply as “p < 0.05”. If available, exact p-values would provide more informative statistical reporting. The authors should also clarify whether any correction for multiple comparisons was considered, given that several modalities are evaluated simultaneously. The formatting of confidence intervals should also be standardized, for example using en dashes consistently rather than mixed hyphen styles.

We have updated Tables 2 and 4 to report exact p-values as $p < 0.001$. As this is a descriptive study evaluating each modality independently against its reference, no multiplicity correction was applied, which is now explicitly stated in the Statistical analysis section. Finally, all confidence intervals in Tables 2 and 4 have been standardized to use en dashes.

15) A careful language and typesetting check is still needed. Several obvious editorial issues remain, such as “reference.Then” without a space, “fingers Sensors positioning”, inconsistent capitalization of “Human Beings”, and phrases such as “presented Table 1”, “As illustrated Fig 6”, or “summarized Table 2”, where “in” is missing. These issues do not affect the scientific meaning but significantly reduce the professional appearance of the manuscript.

We apologize for these editorial oversights. We have thoroughly proofread the manuscript and corrected these typographical errors, including capitalization (e.g., "human beings"), missing spaces, and the addition of missing prepositions ("in") before figures and tables.

16) The reference section requires urgent verification. The manuscript cites references with numbers well beyond 8, including citations such as (12), (18), (19), (20), (23), (24), and (25), whereas the reference list visible in the submitted PDF appears to end at Reference 8 before the figure files begin. The authors should confirm that all cited references are included and that citation numbering is correct throughout the manuscript.

We sincerely apologize for this oversight. The truncation of the reference list in the submitted PDF occurred because we had not updated and synchronized the final bibliography list with our Zotero reference manager after adding several in-text citations. We have now fully updated the bibliography using Zotero, ensuring that all cited references are properly included and that the citation numbering is strictly correct and sequential throughout the manuscript.

17) The reference formatting itself should also be standardized. Some entries contain “[Internet]”, access dates, “Available from:” URLs, and DOI information, whereas other references contain only DOI information. Please carefully check all references against the journal style and ensure consistent formatting of journal abbreviations, dates, page ranges, URLs, and DOIs.

Using Zotero, we applied a consistent journal citation style to ensure uniform formatting across all entries, specifically removing redundant '[Internet]' tags, access dates, and 'Available from' URLs.

18) The manuscript would benefit from a final consistency check of terminology used in figures, tables, and the main text. For example, “PCGS1/PCGS2”, “PCG (S1)/(S2)”, “SCG1/SCG2”, and “SCG (SCG1)/(SCG2)” are used in different locations. A single notation scheme should be adopted throughout all figures, tables, captions, and textual descriptions.

We agree that the terminology was inconsistent. We have now adopted a single, uniform notation scheme throughout the manuscript, figures, and tables: "PCG (S1)", "PCG (S2)", "SCG (SCG1)", and "SCG (SCG2)".

19) The manuscript should also ensure that all mandatory reporting statements are presented clearly and in journal-compliant form. In particular, the ethics/waiver statement should clearly identify the responsible authority or regulatory basis, and the availability of the underlying dataset should be explicitly stated, especially because the Abstract describes the outcome as a “reference dataset”.

We have updated the mandatory reporting statements to fully comply with journal guidelines. The ethics statement in the Methods section has been revised to explicitly cite the regulatory basis for the waiver under French law (Loi Jardé). Furthermore, an explicit "Data Availability Statement" has been added to the manuscript, clarifying that while aggregated data and additional analyses are included in the Supporting Information files, the underlying raw reference dataset is available from the corresponding author upon reasonable request due to institutional data protection policies.

Sincerely,

Pierre Charlier, PhD

Corresponding Author

Attachment Submitted filename: Response_to_Reviewers_auresp_4.docx

# Peer review: Decision Letter 4

**DOI:** 10.1371/journal.pone.0357922.r009

**Authors:** Ming Zhang

24 Aug 2026

<p>Time and Frequency Characteristics of Various Noninvasive Heartbeat Sensors

PONE-D-26-10913R4

Dear Dr. CHARLIER,

We’re pleased to inform you that your manuscript has been judged scientifically suitable for publication and will be formally accepted for publication once it meets all outstanding technical requirements.

Within one week, you’ll receive an e-mail detailing the required amendments. When these have been addressed, you’ll receive a formal acceptance letter and your manuscript will be scheduled for publication.

An invoice will be generated when your article is formally accepted. Please note, if your institution has a publishing partnership with PLOS and your article meets the relevant criteria, all or part of your publication costs will be covered. Please make sure your user information is up-to-date by logging into Editorial Manager at Editorial Manager® and clicking the ‘Update My Information' link at the top of the page. For questions related to billing, please contact billing support.

If your institution or institutions have a press office, please notify them about your upcoming paper to help maximize its impact. If they’ll be preparing press materials, please inform our press team as soon as possible -- no later than 48 hours after receiving the formal acceptance. Your manuscript will remain under strict press embargo until 2 pm Eastern Time on the date of publication. For more information, please contact onepress@plos.org.

Kind regards,

Ming Zhang

Academic Editor

PLOS One

Additional Editor Comments (optional):

Dear Authors,

Thank you for carefully revising your manuscript and addressing the reviewers’ comments.

I am pleased to inform you that the revised manuscript has been accepted for publication. Congratulations, and thank you for choosing our journal for your work.

Best regards,

Academic Editor

Reviewers' comments:

Reviewer's Responses to Questions

**Comments to the Author**

Reviewer #3: All comments have been addressed

**********

2. Is the manuscript technically sound, and do the data support the conclusions?

Reviewer #3: (No Response)

**********

3. Has the statistical analysis been performed appropriately and rigorously?

Reviewer #3: (No Response)

**********

4. Have the authors made all data underlying the findings in their manuscript fully available?

The PLOS Data policy

Reviewer #3: (No Response)

**********

5. Is the manuscript presented in an intelligible fashion and written in standard English?

Reviewer #3: (No Response)

**********

Reviewer #3: (No Response)

**********

what does this mean?). If published, this will include your full peer review and any attached files.

If you choose “no”, your identity will remain anonymous but your review may still be made public.

**Do you want your identity to be public for this peer review?**  For information about this choice, including consent withdrawal, please see our Privacy Policy

Reviewer #3: No

**********

# Editor report: Acceptance letter

**DOI:** 10.1371/journal.pone.0357922.r010

**Authors:** Ming Zhang

PONE-D-26-10913R4

PLOS One

Dear Dr. CHARLIER,

I'm pleased to inform you that your manuscript has been deemed suitable for publication in PLOS One. Congratulations! Your manuscript is now being handed over to our production team.

At this stage, our production department will prepare your paper for publication. This includes ensuring the following:

* All references, tables, and figures are properly cited

* All relevant supporting information is included in the manuscript submission,

* There are no issues that prevent the paper from being properly typeset

You will receive further instructions from the production team, including instructions on how to review your proof when it is ready. Please keep in mind that we are working through a large volume of accepted articles, so please give us a few days to review your paper and let you know the next and final steps.

Lastly, if your institution or institutions have a press office, please let them know about your upcoming paper now to help maximize its impact. If they'll be preparing press materials, please inform our press team within the next 48 hours. Your manuscript will remain under strict press embargo until 2 pm Eastern Time on the date of publication. For more information, please contact onepress@plos.org.

You will receive an invoice from PLOS for your publication fee after your manuscript has reached the completed accept phase. If you receive an email requesting payment before acceptance or for any other service, this may be a phishing scheme. Learn how to identify phishing emails and protect your accounts at https://explore.plos.org/phishing.

If we can help with anything else, please email us at customercare@plos.org.

Thank you for submitting your work to PLOS One and supporting open access.

Kind regards,

PLOS One Editorial Office Staff

on behalf of

Dr. Ming Zhang

Academic Editor

PLOS One
